# Genome-Wide Identification and Genetic Variations of the Starch Synthase Gene Family in Rice

**DOI:** 10.3390/plants10061154

**Published:** 2021-06-06

**Authors:** Hongjia Zhang, Seong-Gyu Jang, San Mar Lar, Ah-Rim Lee, Fang-Yuan Cao, Jeonghwan Seo, Soon-Wook Kwon

**Affiliations:** 1 Department of Plant Bioscience, College of Natural Resources and Life Science, Pusan National University, Miryang 50463, Korea; hjzhangedu@outlook.com (H.Z.); sgjang0136@gmail.com (S.-G.J.); sanmarlar2010@gmail.com (S.M.L.); aar5430@gmail.com (A.-R.L.); NO.1lvtu@outlook.com (F.-Y.C.); rightseo@hotmail.com (J.S.); 2 Life and Industry Convergence Research Institute, Pusan National University, Miryang 50463, Korea

**Keywords:** starch synthase, OsSS, OsGBSS, rice starch, haplotype

## Abstract

Starch is a major ingredient in rice, and the amylose content of starch significantly impacts rice quality. OsSS (starch synthase) is a gene family related to the synthesis of amylose and amylopectin, and 10 members have been reported. In the present study, a synteny analysis of a novel family member belonging to the OsSSIV subfamily that contained a starch synthase catalytic domain showed that three segmental duplications and multiple duplications were identified in rice and other species. Expression data showed that the OsSS gene family is involved in diverse expression patterns. The prediction of miRNA targets suggested that OsSS are possibly widely regulated by miRNA functions, with miR156s targeted to OsSSII-3, especially. Haplotype analysis exhibited the relationship between amylose content and diverse genotypes. These results give new insight and a theoretical basis for the improved amylose content and eating quality of rice.

## 1. Introduction

Rice (Oryza sativa L.) is a staple crop and provides energy for half of the global population [1]. With the economic development and improvement in living standards of the last few decades, consumers have become more concerned about the quality of their food. Important traits associated with quality in rice are nutritional quality, grain appearance, and amylose content [2,3]. 

Starch is the major carbohydrate in rice grains, of which approximately 18% is amylose and 82% is amylopectin [4]. The ratio of amylose to amylopectin plays an important role in the rice grain structure, appearance, and eating quality. The normal percentage of amylose content in the endosperm of rice is classified as waxy (0–2%), very low (2–10%), low (10–20%), intermediate (20–25%), or high (25–33%) [5,6,7]. Amylose and amylopectin have different branched glucose polymers, each of them connected by α-1-4 glycoside and α-1-6 glycoside [8]. Amylose has a lower molecular weight than amylopectin and a small number of long-chain branches, whereas amylopectin has a large number of short-chain branches [9]. Recent research has reported on the genetics and biochemistry of starch biosynthesis. Starch biosynthesis is controlled by adenosine 5’ diphosphate glucose pyrophosphorylase (AGPase), which is a key enzyme governing quality in rice [10]. In addition, seed weight and starch content are increased by overexpression of AGPase genes [11]. 

The synthases for starch biosynthesis have been reported in a previous study [12], including two members of granule bound starch synthase (GBSSI and GBSSII) and four subfamilies of starch synthase (SSI, SSII, SSIII, and SSIV). Each function of GBSSs and SSs in the rice contributes to elongated amylose and amylopectin synthesis [13,14]. Among these, GBSSI, encoded by the waxy gene that regulates the developing seed, is responsible for the biosynthesis and amount of amylose in the rice endosperm [15], whereas GBSSII regulates the biosynthesis of amylose in leaves [16]. The isoforms of SS were also reported to be responsible for the production of amylopectin in the rice endosperm, and relative isoforms were found on the plant tissues. SSI plays the largest role in the total SS activity, accounting for over 60% of this activity in the developing rice endosperm. Furthermore, this gene is expressed anywhere as endosperm and several other cereal tissues [17,18]. SSII-1 and SSIII-2 (SSIIIa) are preferentially expressed in the endosperm, whereas SSII-2, SSII-3, and SSIII-1 (SSIIIb) are mainly expressed in the leaves [19,20].

In the present study, new OsSS members are identified, and the phylogenetic relationships, related expression, miRNA targets, and haplotypes are analyzed, aiming to support the improvement in eating quality in future rice breeding.

## 2. Results

### 2.1. Identification of SS Genes in Rice Genome

**Figure 1:**
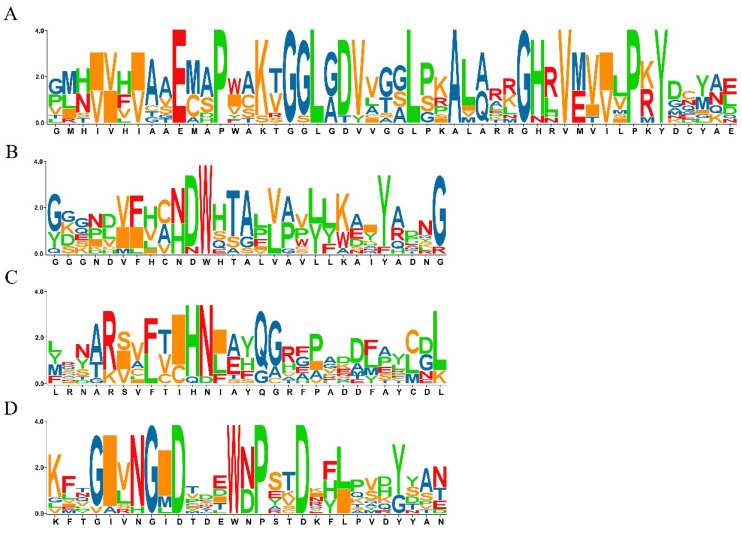
List of putative motifs of the OsSS gene family. (**A**) motif 1; (**B**) motif 2; (**C**) motif 3; (**D**) motif 4. The size of letters represents the similarity of amino acids during multiple sequence alignments, while a single letter indicates that the amino acid is completely conserved.

In order to identify members of SS gene family, the Glyco_transf_5, SS catalytic domain (PFAM accession no. PF08323) was used as the trigger and searched for in the Pfam database, with a filtered E-value set to less than 1E-10. In total, 11 SS genes were identified in the rice genome by HMM software (Table S1); of these, 10 corresponded with the previous study [12]. The gene LOC_Os02g56320, which encodes glycogen synthase 1, was a novel gene showing a significant E-value (3.1E × 10^−34^) in the present study, which suggests that this gene has functions related to starch development in rice. Additionally, a conserved motifs analysis was performed, and four conserved motifs were identified for all SS genes (Figure 1A–D). Through a comparison by position, motifs 1–3 were identified as components of the starch synthase catalytic domain.

In addition, the physical and chemical characteristics of SS genes were analyzed. The genome length of genes ranged from 4981 bp to 11,263 bp, with an average of 7790 bp; the corresponding length of CDS regions ranged from 1827 bp to 5586 bp, with an average of 2675 bp. The computed isoelectric points of these proteins ranged from 4.96 to 6.26, with an average of 5.75; the molecular mass varied from 58 kDa to 205 kDa and averaged 96 kDa (Table 1). For subcellular localization, each protein was predicted by multiple components and positions (Table 2); among these, six proteins were predicted to be located at the chloroplast, and five proteins were predicted to be located in the cytoplasm. 

These results suggested that the starch synthase catalytic domain is a highly conserved domain distributed in SS genes; these acidic nature proteins possible through chloroplast, cytoplasmic, or transferred positions to performed function by protein level.

### 2.2. Analysis of Phylogenetic Relationship and Gene Structure

**Figure 2:**
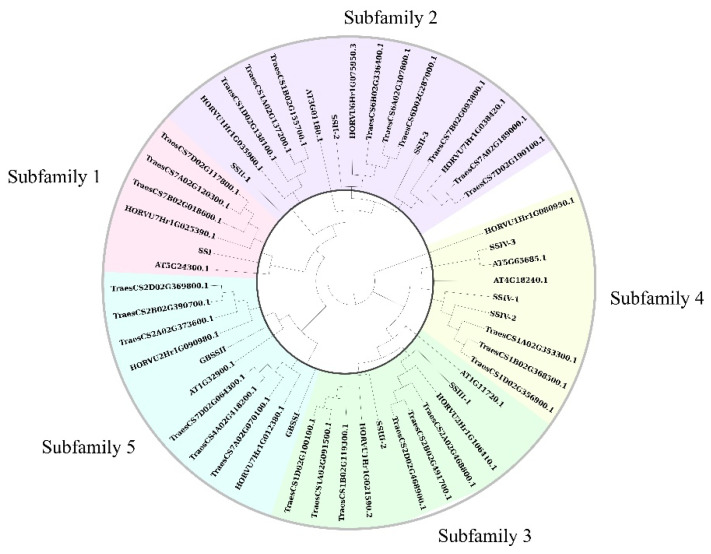
Phylogenetic tree of SSs between rice and other species.

The phylogenetic relationship is crucial to understand the structure of the gene family and the evolutionary history of multiple plant species. In this study, we constructed a phylogenetic tree for SS genes by comparing multiple species, including rice, Arabidopsis, barley, and wheat. Six, nine, and twenty-seven SS genes were identified in three species by the HMM model (the same as described above). The results shown in Figure 2 are based on the phylogenetic relationships. Ten SS genes of rice were consistent with the previous study, so their names were kept the same as previously, divided into five subfamilies according to the grouping results. Additionally, novel family member LOC_Os02g56320 was shown to be closest to SSIV-1 and SSIV-2 and, thus, was designated as SSIV-3, belonging to subfamily 4. In other species, at least one family member existed in each subfamily; only one gene family member existed in subfamilies 1, 2, 3, and 5 of Arabidopsis; and the family member number was the same between rice and barley, while wheat showed more family members in each subfamily.

**Figure 3:**
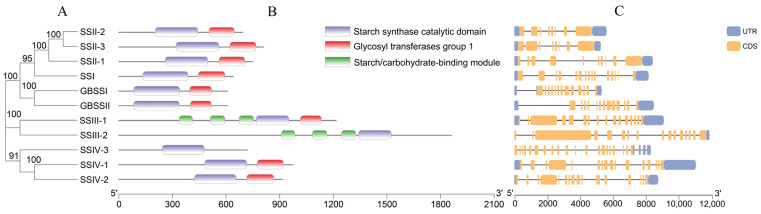
The OsSS gene family structure based on a phylogenetic tree. (**A**) Phylogenetic relationship of OsSS; (**B**) prediction of domain structure; and (**C**) gene structure. The numbers below A and B represent the length of the amino acid (aa) and target sequence (bp), respectively.

Based on the phylogenetic tree, the domain identification and gene structure of OsSS genes were analyzed (Figure 3A). A total of three domains were identified in 11 members (Figure 3B); among these, all members contained the starch synthase catalytic domain, most contained the glycosyltransferases group 1 domain, and only SSIII-1 and SSIII-2 contained a starch/carbohydrate-binding module. This had a significantly different protein length compared with the others, and only the starch synthase catalytic domain existed in protein SSIV-3. In terms of the gene structure, all of the UTR and CDS structures are as shown in Figure 3C, and each family member contained multiple exons in the genomic DNA region.

These results show that SS genes exist in multiple plant species, and the structure of rice shows a closer relationship with barley, which implies that a similar biofunction exists. All of the family members may be involved in starch development, although there is also contained conservative evolution within each subfamily.

### 2.3. Synteny Analysis of SS Genes between Rice and Other Species

**Figure 4:**
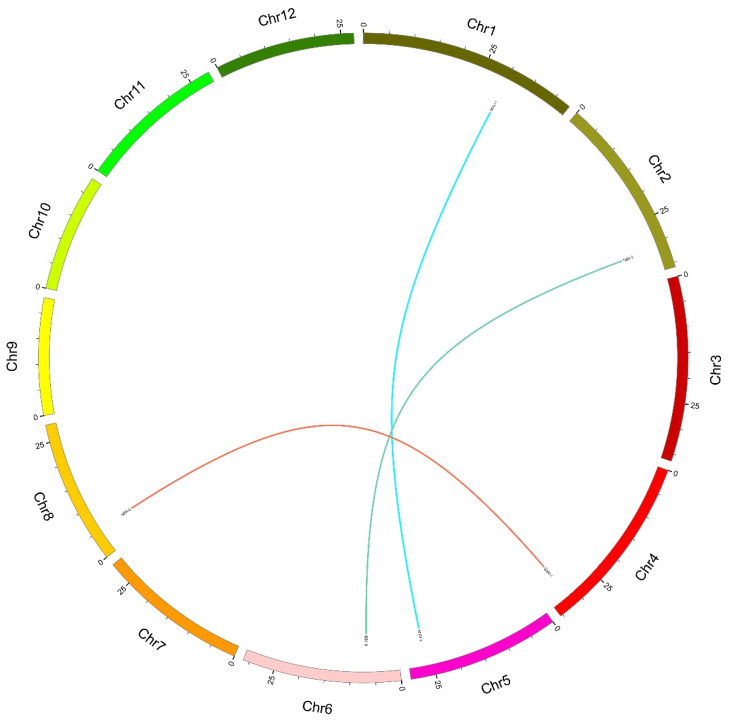
Synteny analysis of *SS* genes in the rice genome. Each rice chromosome is displayed in a different color. Duplicated gene pairs are displayed and linked using lines with that color.

Synteny analysis, such as tandem duplications and segmental duplications, plays an important role in the evolutionary process of a gene family, which could explain the gene variation among diverse species’ genomes. Only the results of genes with pair ≥ 70% sequence identity were considered tandem or segmental duplications. First, we performed a synteny analysis within the rice genome (Figure 4), and the results showed three pairs of segmental duplications. As expected, all segmental duplications corresponded with the subfamily grouping: subfamily 2, SSII-2 and SSII-3; subfamily 3, SSIII-1 and SSIII-2; subfamily 4, SSIV-1 and SSIV-2; and subfamily 5 did not have a duplication event. In order to describe the type and extent of selective pressure during the process of genome duplication, we analyzed the Ka/Ks ratios of all segmental duplication pairs in OsSS genes. Table 3 shows three gene pairs with Ka/Ks ratios ranging from 0.248 to 0.333, with an average of 0.285, which suggests that these gene pairs are involved in the strong purifying/negative selection pressure during evolution in rice.

Moreover, we analyzed duplication events of SS genes between rice and six prevalent plant species, including *Arabidopsis*, barley, maize, sorghum, soybeans, and wheat (Tables S2–S6), using the same criteria as above. There was no duplication event between rice and *Arabidopsis* for *SS* genes (Figure S1A), while two duplication gene pairs were identified with soybeans (Figure S1E and Table S5), and a total of 9, 14, and 14 duplication gene pairs were identified with barley, maize, and sorghum, respectively (Figure S1B–D and Tables S2–S4). Furthermore, a total of 28 duplication gene pairs was detected between rice and wheat (Figure S1F and Table S6). The Ka/Ks ratios between rice and other species were also calculated, showing ranges of 0.12 to 0.42, 0.12 to 0.34, 0.14 to 0.36, 0.08 to 0.18, and 0.1 to 0.38 for the barley, maize, sorghum, soybean, and wheat genomes, respectively. All of the average Ka/Ks ratios for the six species were less than 0.26, suggesting that all of these homologous gene pairs, consistent with the results of segmental duplications in rice, participated in purifying/negative selection pressure in the evolutionary process.

### 2.4. Comprehensive Analysis of the Expression Profiles of SS Genes

**Figure 5:**
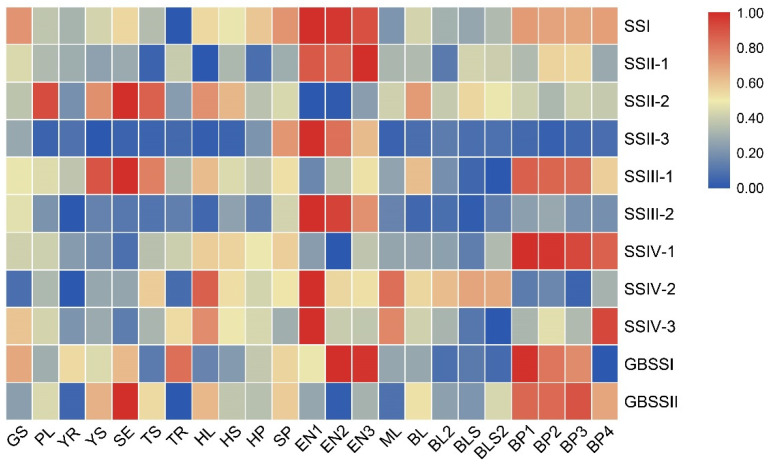
Expression profiles of OsSS genes in diverse tissues and across different stages. Expression Data (FPKM) standardization in each line and re-count relative expression from 0 to 1 was performed. GS: germinating seed; PL: plumule; YR: radicle; YS: young seedling; SE: seedling at trefoil stage; TS: shoot under 2 tillers; TR: root under 2 tillers; HL: flag leaf in heading date; HS: stem in heading stage; HP: panicle in heading stage; SP: spikelet; EN1: endosperm seven days after pollination; EN2: endosperm 14 days after pollination; EN3: endosperm 21 days after pollination; ML: flag leaf in mature stage; BL: mature leaf blade under young panicle; BL2: mature leaf blade under mature panicle; BLS: mature leaf sheath under young panicle; BLS2: mature leaf sheath under mature panicle; BP1: developing panicle (length < 1 mm); BP2: developing panicle (3 mm < length < 5 mm); BP3: developing panicle (10 mm < length < 15 mm); BP4: developing panicle (40 mm < length < 50 mm).

RNA-seq data from different tissues and growth stages were analyzed for relative expression to understand the expression pattern of OsSS genes. The results are shown in Figure 5. The expression of family members varied between tissues and growth stages, but some genes generated a similar grouping phenomenon or similar expression in one or a few tissues simultaneously. Among these, SSI, SSII-1, SSII-3, SSIII-2, and GBSSI showed the highest expression in the endosperm development stage (EN1–EN3). The expressions of SSIV-2 and SSIV-3 were the highest in EN1 but decreased in EN2–EN3, while SSIII-1, SSIV-1, GBSSI, and GBSSII showed higher or the highest expression in the panicle development stage. Interestingly, SSII-3 and SSIII-2 showed inactive expression in most tissues except in the endosperm development stage. These results suggested that few genes possible are involved in the grouping expression pattern, and they performed distinctive functions in different tissues and growth stages.

Generally, the tandem and segmental duplications showed similar expression patterns that covered the whole growth stage in plants [21]. Due to three pairs of segmental duplications being found in this family (Figure 4), the expression pattern following the growth stage of three gene pairs was compared (Figure S2). The results showed that the expression variation in three gene pairs did not have a similar pattern, but combined with previous results (Figure 5), SSIII-2 and SSII-3 showed similar variation, with increased expression in the flowering stage (Figure S2A,B). This implies that, although SSIII-1 and SSIII-2, SSII-2, and SSII-3 involved segmental duplications, these possibly had different functions in plants, whereas SSII-3 and SSIII-2 possibly had similar functions for starch development. 

Moreover, we analyzed other RNA-seq data for a further functional understanding of OsSS genes. The endosperm-specific results in Figure S3A show two subgroups divided due to expression variation in different tissues. SSIV-1, GSBBII, SSII-2, and SSIII-1 had higher expression in the ovaries and embryo (Figure S3A), while other genes showed higher expression in the endosperm during the endosperm development stage. Additionally, the RNA-seq results of starch-related genes mutant were analyzed in the OsbZip58-1 mutant line, and only the expression of GBSSI and SSII-1 showed repressed expression (Figure S3B). In the gif1 mutant line, GBSSII and SSIII-1 were activated by mutation of gif1, while SSII-1 and SSIV-3 showed decreased expression (Figure S3C). These results implied that some SS genes are possibly involved in signaling pathways through interaction with other starch-related proteins, thus performing the function of regulating starch development in rice.

### 2.5. Prediction of Regulation Network by miRNA-Targeted SS Genes

miRNA has crucial functions in the processes of plant growth, metabolism, signal transduction, etc. We analyzed the potential miRNA targets of 11 SS genes. The results showed 80 unique putative target pairs identified with mature miRNAs of 19–24 nucleotides long by the rice database (Table S7), and all SS family members were identified as containing putative target miRNAs. There were two major regulation networks found (Figure S4), and at least one family member was identified in each subfamily. All members of subfamily 4 contained major miRNA targets. Interestingly, the miRNAs involved in targeting SSII-3 showed 16 target pairs, most (11 of 16) of which belonged to the miRNA156 class, only 5 of 16 pairs belonged to other miRNAs. Additionally, of the 80 target pairs, 68 were shown to regulate cleavage, while only 12 regulated translation. These results implied that the miRNA156-performed starch synthesis-related function might be regulated by SSII-3 and that cleavage function as the major type for miRNA performed this regulation with target genes in the starch-related process.

### 2.6. Haplotype Analysis for OsSS Genes

**Figure 6:**
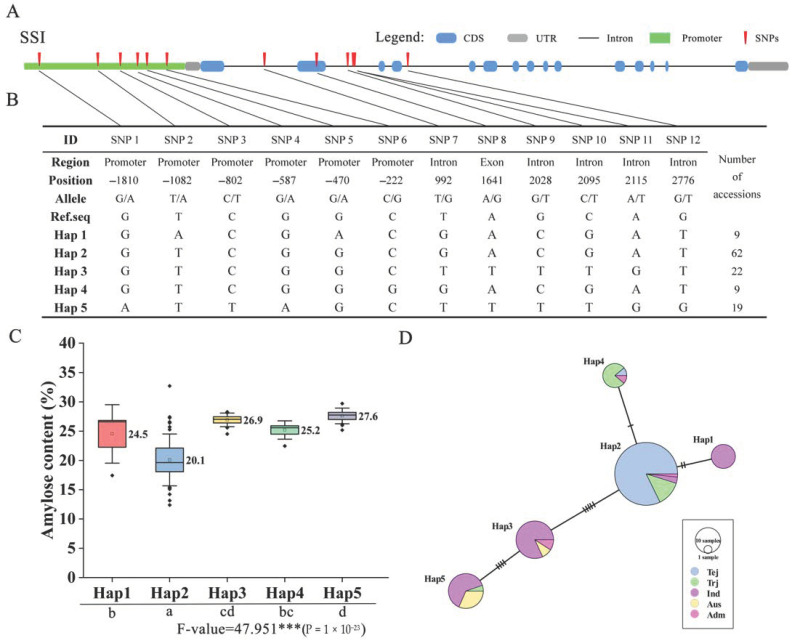
Haplotype analysis of *OsSSI*. (**A**) Structural representation of *OsSSI* and upstream promoter region. (**B**) *OsSSI* SNPs and haplotype groups in 137 rice accessions. SNP positions are given relative to the start of the 5′UTR. Hap: haplotype. (**C**) Association of phenotype with haplotype. Different letters indicate significant AC differences between haplotypes (ANOVA, Duncan test). (**D**) Haplotype network variation of *OsSSI*. The circle size represents the number of accessions in each Hap, and the number of transverse lines between each Hap represents the number of nucleotide variations. Tej: Temperate *japonica*; Trj: Tropical *japonica*; Ind: *Indica*; Aus: *Aus*; and Adm: Admixture rice varieties.

In rice, some of the SS genes’ functions have been reported [13,22,23], and diverse haplotypes or alleles that impacted the starch-related index were identified in multiple varieties [24]. In the present study, we also identified novel haplotypes for amylose content using a core collection set. After we removed the heterozygotes and missing data, the SNPs located in the promoter, UTR, exon, and intron region were used for the haplotype and haplotype variation analysis. For analysis of SSI, 12 SNPs were identified in the promoter, intron, and exon regions (Figure 6A), and five Haps were generated by those SNPs (Figure 6B). A boxplot showed an association of five Haps and AC phenotypes (Figure 6C), with different significance levels decided by ANOVA (Duncan test). Among these, Hap 2 contained the lowest AC compared with other Haps, with an average of 20.1%. Hap 1 and Hap 4 contained a moderate level of AC, with averages of 24.5% and 25.2%, respectively. Hap 3 and Hap 5 showed the highest AC in this population, with averages of 26.9% and 27.6%, respectively. Moreover, we analyzed the haplotype variation network between each Hap, which showed that the five Haps were separated roughly into two subgroups (Figure 6D). Hap 2 and Hap 4 possessed mostly Tej and Trj and showed a close relationship; there was only an alteration of one SNP. Hap1, Hap3, and Hap5 possessed mostly Ind and Adm varieties, forming a subgroup with distant genetic relationships with Japonica, though there were alterations of multiple SNPs between each pair of Haps. For analysis of SSIV-2, GBSSI, and GBSSII, there were six Haps generated by 5, 9, and 17 SNPs of diverse regions (Figure S5A,B, Figure S6A,B, Figure S7A,B). Similar to SSI, there were two major groups (Jap and Ind) in GBSSI and GBSSII (Figure S6D and 7C), but in SSIV-2, Hap 4 and Hap 5 were mixed in different varieties (Figure S5C). In terms of associations with phenotype, the lowest and highest Haps were identified, including Hap 2 and Hap 6 of SSIV-2 (Figure S5D), Hap 3 and Hap 6 of GBSSI (Figure S6C), and Hap 6 and Hap 2 of GBSSII (Figure S7D). Interestingly, there was an SNP (–1596 bp) in the promoter region of SSIV-2, which produced a nucleotide mutation from C to T. By comparison, it is possible that a key mutation made a major contribution to AC, as there was a significant difference in two genotypes that possessed AC averages of 20.7 and 26 (Figure S5E). Similarly, a key SNP was found in GBSSII, showing a nucleotide change from T to G at the –800 bp position, which produced phenotype variations from 26.4 to 20.8, a highly significant difference (Figure S7E). Taken together, these haplotypes of SSI, SSIV-2, GBSSI, and GBSSII showed that the functions involved AC in rice and thus impacted starch-related development; these results support the theoretical basis for the preferable selection of rice eating quality.

## 3. Discussion

In plants, starch-related traits are regulated by multiple gene family members, including ADP-glucose pyrophosphorylases (AGPs), starch branching enzymes (SBEs), starch degradation enzymes (DBEs), and starch synthases (SSs) [24]. A series of starch and metabolic processes are generated by the interactions of these biosynthetic enzymes [25]. Among these, the SS family is involved in the regulation of the structure of amylose and amylopectin [26], the content of each [27], the physical and chemical properties of starch [28], the gelatinization temperature [29], etc., and was critical to starch synthesis and eating quality in rice [30]. A previous study identified 10 SS genes belonging to rice by a BLAST database, divided into five subfamilies according to the phylogenetic relationship [12]. In the present study, we used the PFAM database and HMM model (PF08323), searched the rice genome, and identified 11 genes by their significantly low E-values. The novel family member LOC_Os02g56320, a biosynthetic enzyme as glycogen synthase 1, had an E-value of 3.10 × 10^−34^ (Table S1), suggesting that its functions might be starch-related. Through a phylogenetic study, we found that this novel member was most closely related to SSIV-1 and SSIV-2 and clustered with subfamily 4 in other species; thus, we named it SSIV-3, a novel member of subfamily 4 in the OsSS gene family (Figure 2). Interestingly, we searched the SS gene family in the Arabidopsis TAIR database and found gene AT4G18240 (named AtSS4 (AtSSIV)) and gene AT5G65685 (named AtSS5), which belong to the SS gene family of Arabidopsis. In our results, SSIV-3 was most closely related to AtSS5, while SSIV-1 and SSIV-2 are closer to AtSS4; thus, SSIV-3 was the novel family member verified by a phylogenetic study between rice and Arabidopsis. Furthermore, we analyzed the gene’s and domain’s structure, which showed that a starch synthase catalytic domain existed in all family members, and a glycosyl transferases group 1 existed in most members except SSIII-2 and SSIV-3. The starch/carbohydrate-binding module only existed in the SSIII subfamily; these results present the new gene SSIV-3 as a member of the OsSS family due to the starch synthase catalytic domain. This domain might be a major domain that performs starch-related functions.

A synteny analysis is important for detecting duplication events during the evolutionary process in diverse species [31]. The Ka/Ks ratio supports a deep understanding of the type and degree of selection pressure between duplications (tandem and segmental) [32]. Therefore, we performed a synteny analysis of the OsSS gene family and compared it with the other six genomes. This showed three segmental and no tandem duplications were found intra rice genome, represented in subfamily SSII, SSIII, SSIV, and possibly contained duplications events. Among these, OsSSII-3 (named ALK) was involved in the regulation of the gelatinization temperature of starch [29], OsSSIII-2 (named flo5 by mutant identification) impacted multiple characteristics of starch [23], and OsSSIV-1 showed no significant function due to a single mutant, but mutation in cooperation with flo5 produced spherical starch granules [13]. In combination with the present study, SSII-2, SSIII-1, and OsSSIV-2 also possibly involved a similar function of duplication homologs. In addition, 0, 9, 14, 14, 2, and 28 duplications were identified between the rice and Arabidopsis, barley, maize, sorghum, soybean, and wheat genomes, respectively. These results suggest a functional differentiation between rice and Arabidopsis SS genes, with wheat as the closest species to compare with the other five genomes. SS genes might be involved in similar functions in starch synthases. 

Expression analysis enables researchers to understand gene function at the transcriptional level. With the development of sequencing technology, numerous RNA-seq (transcriptome analysis) results related to starch function have been reported constantly for the integrated analysis of expression variation in the whole genome. In the present study, we used RNA-seq data to gain a deep understanding of the expression mechanisms of the OsSS gene family and found a grouped expression pattern for the whole family. SSI, SSII-1, SSII-3, SSIII-2, and GBSSI showed significantly higher expression in the endosperm development stage, while SSIII-1, SSIV-1, GBSSI, and GBSSII showed higher expression in the panicle and SSIV-2 and SSIV-3 exhibited expression in the earlier stage of endosperm development (Figure 6). In other RNA-seq results, there was also grouping into two groups by the expression patterns in the ovaries, embryo, and endosperm (Figure S3). These results suggest that OsSS genes are possibly involved in a synergy pattern and participate in starch-related signaling pathways in different stages. On the other hand, the regulation pathway of rice starch synthesis has been reported in recent years, with Osbzip58 as a transcription regulator showing interaction and redundancy function with RPBF (rice prolamin box binding factor). It could also interact with the protein OsLOL1, activate the expression of OsKO2, and/or stimulate aleurone programmed cell death through an impact on GA biosynthesis [33,34]. In another study, an OsSSIIa/OsSSIIIa double mutant did not have an additive effect in rice, possibly due to the interaction with amylophosphorylase [22]. In the present study, we checked the expression change in mutant RNA-seq data. In the OsbZip58-1 mutant, SSII-1 and GBSSI decreased in comparison with other SS genes. SSII-1 and SSIV-3 showed a decrease in the gif-1 mutant. These results suggest that SSII-1, GBSSI, and SSIV-3 are possibly involved in starch synthesis by a regulation mechanism. Additionally, miRNA could regulate target genes’ expression by a cleavage and translation model [35]. We also engaged in the prediction of the miRNA regulation network between OsSS genes and the published OsmiRNAs. Two networks were identified, and a miRNA156s-SSII-3-specific pathway was found (Figure S4), with cleavage shown in almost all inhibition functions. These results showed that OsSS genes might be involved in transcription regulation by miRNA target, with cleavage as a major function in miRNA–SS gene target regulation.

Haplotype analysis showed the importance of understanding diverse genotype functions in diverse varieties [36]. In OsSS genes, some gene haplotypes have already been reported, including the positive or negative influence on rice starch-related traits, such as Waxy gene impacts on AC and SSII-3 gene impacts on gelatinization temperature [24]. In the present study, we performed a haplotype analysis of four genes to detect the effects on variations of AC; each significant variation was identified by the phenotype associated with diverse Haps. Especially given the haplotype network results, we could speculate on the origin of haplotype evolutionary relationships. For example, in SSI, Hap 2 contained a small number of SNP variations with Hap 4 (2) and Hap 1 (2) but had a large number of SNP variations with Hap 3 (5) and Hap 5 (9) (Figure 6D). Based on the ingredients of these Haps, we speculated that Jap of Hap 4 and Ind of Hap 1 were derived from Hap 2, and Hap 3 was possibly also derived from Hap 2 but produced large variations. Hap 5 seemed to be derived from Hap 3. Similarly, the presence of major origin Haps could also be speculated for Hap 2 in GBSSI (Figure S6D), and Hap 4 and Hap 5 in GBSSII (Figure S7C), derived other minor Haps. Additionally, the key SNP was found in SSIV-2, where Hap 2, Hap 3, and Hap 4 possessed lower AC compared to other Haps (Figure S5E). As expected, genotype C of Hap 2, Hap 3, and Hap 4 have performed grouping, unlike with T of Hap 1, Hap 5, and Hap 6 (Figure S5B). These results suggest that key SNPs possibly played a decisive role in AC.

## 4. Materials and Methods

### 4.1. Identification of SS Genes in the Rice Genome

For the identified SS family members in rice, the rice reference genome was obtained from the resource database Phytozome (phytozome.jgi.doe.gov), and a Hidden Markov Model (HMM) search was conducted via the HMMER 3.0 program [37], with the objective model Glyco_transf_5 (Pfam accession: PF08323) as a query for the HMM search that was obtained from the Pfam database (http://pfam.xfam.org last accessed on 6 June 2021) [38]. The E-value threshold was set to less than 10-10 for selected candidates, with the candidate domain again searched on the Pfam data for the presence of the Starch synthase catalytic domain after the retrieval. For conserved motif identification, meme software was used for finding a specific motif and positions in SS genes (http://meme-suite.org, last accessed on 6 June 2021) [39]. The following parameters were set: motif length, 6 to 50, zero or one occurrence per sequence (ZOOPS), and a maximum of four motifs. For the analysis of physical and chemical characteristics, all protein sequences were uploaded and analyzed in ExPasy website tools (https://www.expasy.org, last accessed on 6 June 2021). For the predicted subcellular localization of each member, all protein sequences were analyzed by website tools CELLO v.2.5: subCELlular LOcalization predictor [40], through a comparison of the results of prediction for diverse positions. Only the position that contained the maximum value of prediction was decided as the final position. 

### 4.2. Phylogenetic and Structure Analysis of OsSS Genes

For the phylogenetic analysis, a phylogenetic tree of rice and other species was generated. First, the query sequences of Arabidopsis, barley, and wheat were confirmed to be consistent with rice through an HMM model search and download from the Ensembl database (http://ensembl.gramene.org, last accessed on 6 June 2021). Afterward, full protein sequences were uploaded into MEGA-X software for sequence alignment and the generation of a NJ tree by 1000 bootstraps [41]. Finally, a Newick file was uploaded into website tools iTOL (https://itol.embl.de, last accessed on 6 June 2021) for the visualization of the circle tree [42]. For gene structure and domain analysis, the integrative toolkit TBtools was used [43], with the rice reference gff3 file and protein name used to construct the gene structure information. Full protein sequences were uploaded into the Pfam database to identify the conserved domain and positions. Based on the phylogenetic relationship, the visualization mixture of the plot was performed using TBtools.

### 4.3. Gene Duplication and Synteny Analysis

All query sequences of the other species were obtained from the Ensembl database based on an HMM model search to analyze the duplication events in OsSS genes. The SS genes’ sequences were found by the Blastp function in BLAST software [44]; afterward, all gene pairs were analyzed for synteny by MCScanX software, following the official procedures [45]. Among these, the identity and query coverage were >70% only for the duplicated gene pair, and tandem duplications were decided by the distance between gene pairs, within 100 kb [46]. Visualization of the circle plot using the software Circos was according to the results of MCScanX [47]. The duplicated gene pairs were connected by a solid line. Synonymous and nonsynonymous nucleotide substitution rates of duplicated gene pairs were calculated using KaKs Calculater 2.0 software [48]. The mode of selection was identified by the Ka/Ks ratio, with Ka = Ks (Ka/Ks =1), Ka < Ks (Ka/Ks <1), and Ka > Ks (Ka/Ks > 1) representing neutral mutation, negative (purifying) selection, and positive (diversifying) selection, respectively, during the two comparisons.

### 4.4. Expression Analysis of SS Genes

RNA-seq analysis was used in the present study to analyze the expression pattern of OsSS genes. Total RNA was extracted from the variety Minghui 63 [49] using Trizol according to the manufacturer’s instructions to analyze the expression variation of different stages and tissues. Through a filtering database, the results of SS genes were selected for analysis, and the data were calculated from three replications. For other RNA-seq data, the expression data from rice embryo and endosperm development were used for reference [50,51], and the data were calculated from two replications. Two expression profiles of mutant materials were used to detect the relationships between SS genes and OsbZip58-1 and gif1 [52]. Heat maps were created using TBtools, and a bar plot was plotted by website tools (www.bioinformatics.com.cn, last accessed on 6 June 2021), an online platform for data analysis and visualization.

### 4.5. Prediction of Regulation Network for miRNA-Target SS Genes 

The miRNA database (http://plantgrn.noble.org, last accessed on 6 June 2021) was used for detecting the miRNA target genes of the SS family [53] To analyze the putative regulation network of SS genes. For rice, 713 miRNAs and the cds sequences of SS genes were part of the analysis, and the results were filtered according to those equal to or less than 4.5, with a plot of relationship network created by the software Cytoscape [54].

### 4.6. Haplotype Analysis for OsSSs

A core collection was used for the present study. The whole panel contained 137 varieties that included subspecies of Temperate and Tropical japonica, Indica, Aus, Aromatic, and Admixture. Information, in the way of high-quality genotype and phenotype data, followed previous reports [55,56]. The haplotype analysis included whole SNP markers from the intragenic and promoter’s region but excluded missing and heterozygote data. The promoter region was set to 2000 bp upstream of the gene initiation site (ATG), and a visualization of the gene structure was produced by website tools Gene Structure Display Server 2.0 (http://gsds.gao-lab.org, last accessed on 6 June 2021) [57]. For the haplotype analysis, the average of the phenotype and varieties number were calculated from the phenotype data of each subspecies. ANOVA analysis and Duncan test were performed by SPSS software after grouping and association, with the plot visualized using boxplot by software Origin. Haplotype variation (Network) was performed by software PopART according to the haplotype analysis results [58].

## 5. Conclusions

In this study, we re-performed the identification of the OsSS gene family. A novel member, SSIV, was found by the HMM model, potential miRNA targets of OsSS genes were identified, and miR156s seemed to be the major miRNA targeted by OsSSII-3. Diverse haplotypes of OsSS genes showed relationships between genotype variations and starch content. Our results will be helpful for improving the starch-related characteristics and eating quality of rice.

## Figures and Tables

**Table 1:** Gene list and information for starch synthase.

Gene	RAP-ID	MSU-ID	Chr.	Start	End	Gene Length	Cds Length	PI	MW
*SSI*	Os06g0160700	LOC_Os06g06560	Chr6	3,079,059	3,086,808	7750	1926	5.71	59,234.43
*SSII-1*	Os10g0437600	LOC_Os10g30156	Chr10	15,673,128	15,681,124	7997	2250	5.39	78,907.22
*SSII-2*	Os02g0744700	LOC_Os02g51070	Chr2	31,232,888	31,238,210	5323	2085	6.04	75,623.36
*SSII-3*	Os06g0229800	LOC_Os06g12450	Chr6	6,748,358	6,753,338	4981	2433	5.28	86,809.55
*SSIII-1*	Os04g0624600	LOC_Os04g53310	Chr4	31,750,955	31,759,581	8627	3651	5.42	137,944.3
*SSIII-2*	Os08g0191433	LOC_Os08g09230	Chr8	5,352,105	5,363,367	11263	5586	4.96	205,368.8
*SSIV-1*	Os01g0720600	LOC_Os01g52250	Chr1	30,030,997	30,041,476	10480	2928	5.93	100,337.5
*SSIV-2*	Os05g0533600	LOC_Os05g45720	Chr5	26,485,807	26,494,112	8306	2748	6.03	104,178.5
*SSIV-3*	Os02g0807100	LOC_Os02g56320	Chr2	34,475,930	34,483,804	7875	2163	6.18	81,317.8
*GBSSI*	Os06g0133000	LOC_Os06g04200	Chr6	1,765,622	1,770,656	5035	1830	6.1	58,473.21
*GBSSII*	Os07g0412100	LOC_Os07g22930	Chr7	12,916,277	12,924,325	8049	1827	6.26	67,354.78

PI: Isoelectric point. MW: Molecular weight.

**Table 2:** Prediction of subcellular localization in the starch synthase gene family.

Gene	Comp. Result	Di-pep. Result	part-Comp. Result	chemotype. Result	Neighbor	Extracellular	Plasma Membrane	Cytoplasmic	Cytoskeletal	ER	Golgi	Lysosomal	Mitochondrial	Chloroplast	Peroxisomal	Vacuole	Nuclear	Predicted Location
*SSI*	Chloroplast	Lysosomal	Chloroplast	Extracellular	Chloroplast	0.414	0.399	0.96	0.017	0.155	0.029	0.834	0.503	1.233	0.323	0.048	0.085	**Chloroplast**
*SSII-1*	Cytoplasmic	Chloroplast	Chloroplast	Chloroplast	Chloroplast	0.227	0.078	0.723	0.013	0.076	0.016	0.248	0.752	2.519	0.223	0.057	0.069	**Chloroplast**
*SSII-2*	Cytoplasmic	Chloroplast	Chloroplast	Chloroplast	Chloroplast	0.053	0.015	0.539	0.01	0.071	0.011	0.018	0.441	3.628	0.087	0.081	0.046	**Chloroplast**
*SSII-3*	Cytoplasmic	Chloroplast	Chloroplast	Mitochondrial	Chloroplast	0.135	0.199	0.87	0.032	0.119	0.056	0.037	0.468	2.5	0.186	0.099	0.3	**Chloroplast**
*SSIII-1*	Cytoplasmic	Nuclear	Cytoplasmic	Nuclear	Cytoplasmic	0.079	0.225	2.15	0.043	0.074	0.046	0.009	0.228	0.452	0.076	0.018	1.6	**Cytoplasmic**
*SSIII-2*	Nuclear	Nuclear	Cytoplasmic	Nuclear	Cytoplasmic	0.149	0.523	1.93	0.042	0.142	0.082	0.01	0.1	0.078	0.056	0.024	1.861	**Cytoplasmic**
*SSIV-1*	Cytoplasmic	Cytoplasmic	Cytoplasmic	Nuclear	Cytoplasmic	0.144	0.086	3.034	0.026	0.053	0.116	0.011	0.326	0.274	0.097	0.012	0.82	**Cytoplasmic**
*SSIV-2*	Cytoplasmic	Cytoplasmic	Nuclear	Nuclear	Cytoplasmic	0.11	0.113	2.109	0.066	0.04	0.141	0.013	0.517	0.278	0.116	0.009	1.487	**Cytoplasmic**
*SSIV-3*	Nuclear	Cytoplasmic	Cytoplasmic	Cytoplasmic	Cytoplasmic	0.342	0.288	2.117	0.013	0.046	0.036	0.07	0.567	0.229	0.142	0.014	1.137	**Cytoplasmic**
*GBSSI*	Chloroplast	Chloroplast	Chloroplast	Chloroplast	Chloroplast	0.022	0.039	0.188	0.004	0.006	0.003	0.012	0.558	4.01	0.102	0.023	0.032	**Chloroplast**
*GBSSII*	Cytoplasmic	Chloroplast	Chloroplast	Chloroplast	Chloroplast	0.076	0.177	1.444	0.01	0.042	0.021	0.096	0.25	2.292	0.519	0.029	0.045	**Chloroplast**

**Table 3:** Synteny analysis for the starch synthase gene family in the rice genome.

Homologous Genes in Rice Genome					Homologous Genes in Rice Genome					Ka	Ks	Ka/Ks	S	N
Gene	Gene ID	Chr.	Start	End	Gene	Gene ID	Chr.	Start	End					
*SSIV-1*	LOC_Os01g52250	Chr1	30030997	30,041,476	*SSIV-2*	LOC_Os05g45720	Chr5	26,485,807	26,494,112	0.19382	0.78169	0.24795	295.917	358.083
*SSII-2*	LOC_Os02g51070	Chr2	31232888	31,238,210	*SSII-3*	LOC_Os06g12450	Chr6	6,748,358	6,753,338	0.23731	0.71259	0.33303	236.083	317.917
*SSIII-1*	LOC_Os04g53310	Chr4	31750955	31,759,581	*SSIII-2*	LOC_Os08g09230	Chr8	5,352,105	5,363,367	0.23734	0.86354	0.27484	409.083	568.917

## Data Availability

The data are available in the article and supplementary materials.

## Supplementary Materials

The following are available online at https://www.mdpi.com/article/10.3390/plants10061154/s1, Figure S1: Synteny analysis of SSs between rice and other genomes, Figure S2: Expression profiles of duplications across different growth stages, Figure S3: Expression profiles of *OsSS* genes, Figure S4: Putative miRNA targets of *OsSS* genes, Figure S5: Haplotype analysis of *OsSSIV-2*, Figure S6: Haplotype analysis of *OsGBSSI*, Figure S7: Haplotype analysis of *OsGBSSII*, Table S1: Motif identification based on PFAM database, Table S2: Synteny analysis for starch synthase gene family between rice and barley genome, Table S3: Synteny analysis for starch synthase gene family between rice and maize genome, Table S4: Synteny analysis for starch synthase gene family between rice and sorghum genome, Table S5: Synteny analysis for starch synthase gene family between rice and soybean genome, Table S6: Synteny analysis for starch synthase gene family between rice and wheat genome, Table S7: Prediction of miRNAs targeted OsSSs identified by psRNATarget online tool.

## Abbreviations

NJ	neighbor-joining
Ks	synonymous
Ka	nonsynonymous
SS	Starch synthase
GBSS	granule-bound starch synthase
AC	amylose content
Hap	haplotype
Tej	Temperate Japonica
Trj	Tropical Japonica
Ind	Indica
Adm	Admixture
GA	gibberellins
