# Supplementary material for: Genome-Wide Identification and Genetic Variations of the Starch Synthase Gene Family in Rice
Source: Plants (Basel). 2021 Jun 6;10(6):1154. doi: 10.3390/plants10061154 (PMC8227427; doi:10.3390/plants10061154)

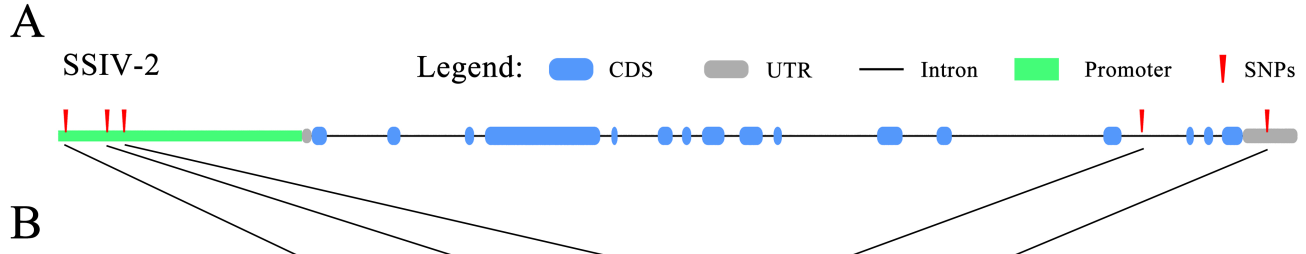

B

ID	SNP 1	SNP 2	SNP 3	SNP 4	SNP 5	Number of accessions
**Region**	Promoter	Promoter	Promoter	Intron	3'UTR	
**Position**	−1936	−1596	−1457	6896	7924	
**Allele**	C/A	C/T	G/A	A/T	G/A	
**Ref.seq**	C	C	G	A	G	
**Hap 1**	A	T	G	A	G	8
**Hap 2**	C	C	A	A	G	6
**Hap 3**	C	C	G	A	A	39
**Hap 4**	C	C	G	A	G	21
**Hap 5**	C	T	G	A	G	6
**Hap 6**	C	T	G	T	G	41

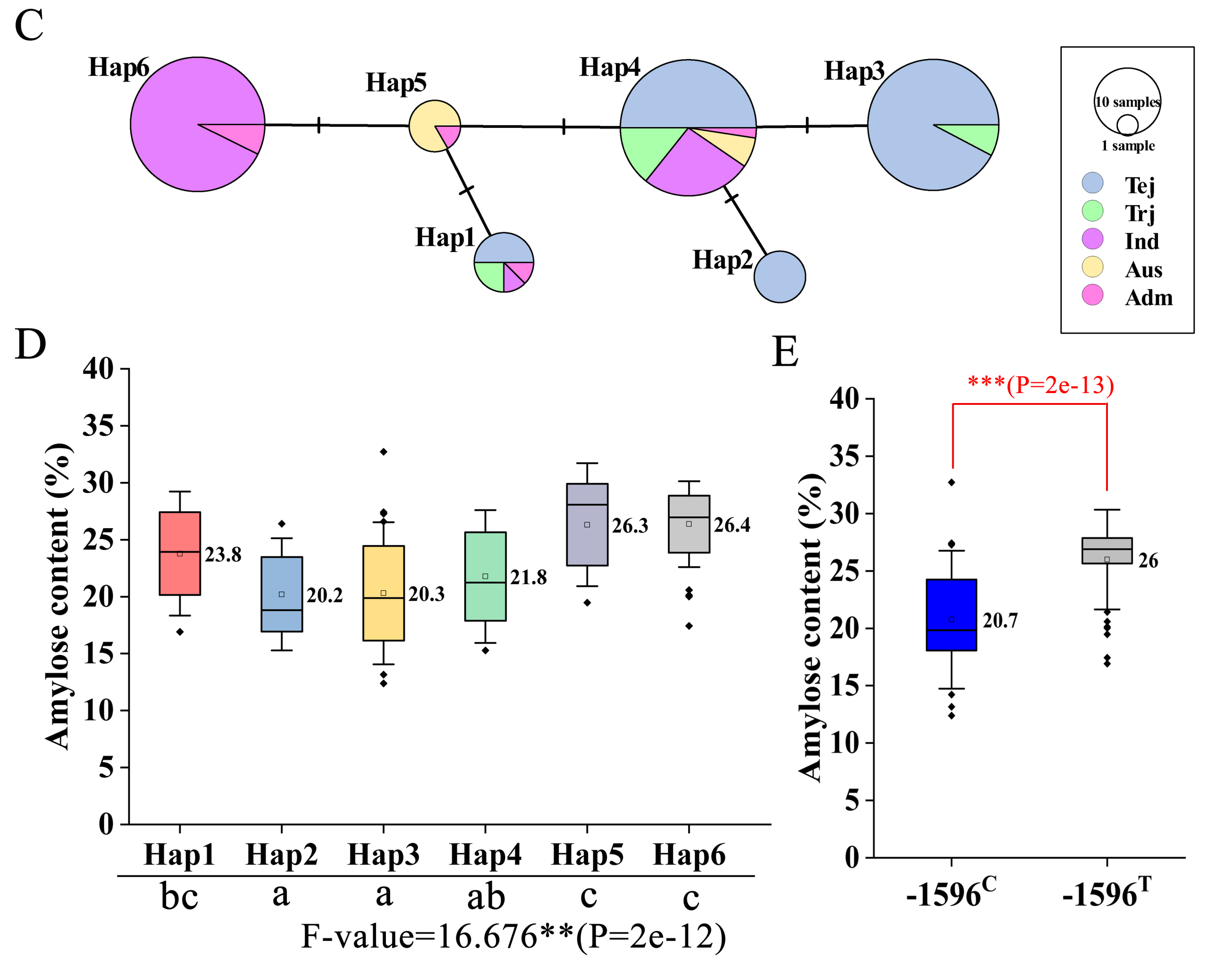

Supplement: Supplementary file 1 [file plants-10-01154-s001.zip › Fig S5.pdf]
