# Supplementary material for: Genome-Wide Identification and Genetic Variations of the Starch Synthase Gene Family in Rice
Source: Plants (Basel). 2021 Jun 6;10(6):1154. doi: 10.3390/plants10061154 (PMC8227427; doi:10.3390/plants10061154)

A

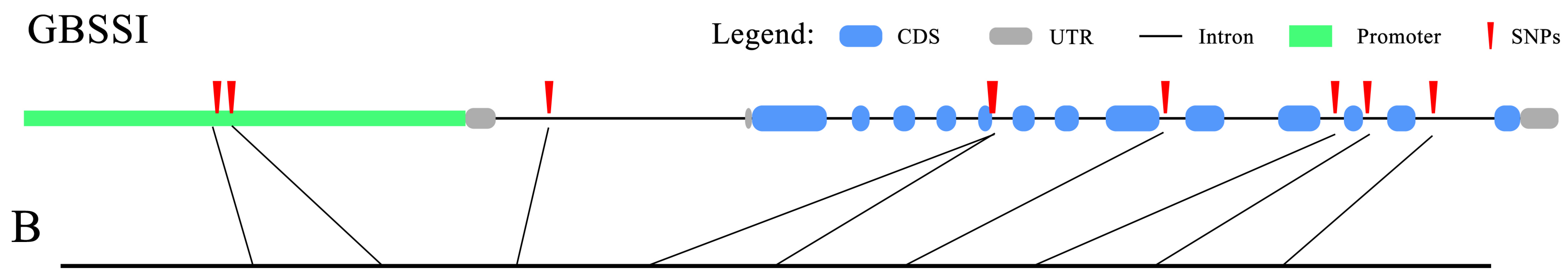

B

ID	SNP 1	SNP 2	SNP 3	SNP 4	SNP 5	SNP 6	SNP 7	SNP 8	SNP 9	
**Region**	Promoter	Promoter	Intron	Intron	Intron	Intron	Intron	Intron	Intron	Number of accessions
**Position**	–1123	–1058	381	2384	2395	3173	3942	4088	4387	
**Allele**	T/C	A/G	C/T	A/C	C/T	A/G	T/C	A/G	T/A	
**Ref.seq**	T	A	C	A	C	A	T	A	T	
**Hap 1**	C	G	C	A	C	G	C	G	T	6
**Hap 2**	T	A	C	A	C	A	T	A	T	55
**Hap 3**	T	A	C	A	C	G	T	A	T	2
**Hap 4**	T	A	C	C	C	A	T	A	T	24
**Hap 5**	C	G	T	A	T	A	T	A	A	26
**Hap 6**	C	G	T	A	T	A	T	A	T	6

C

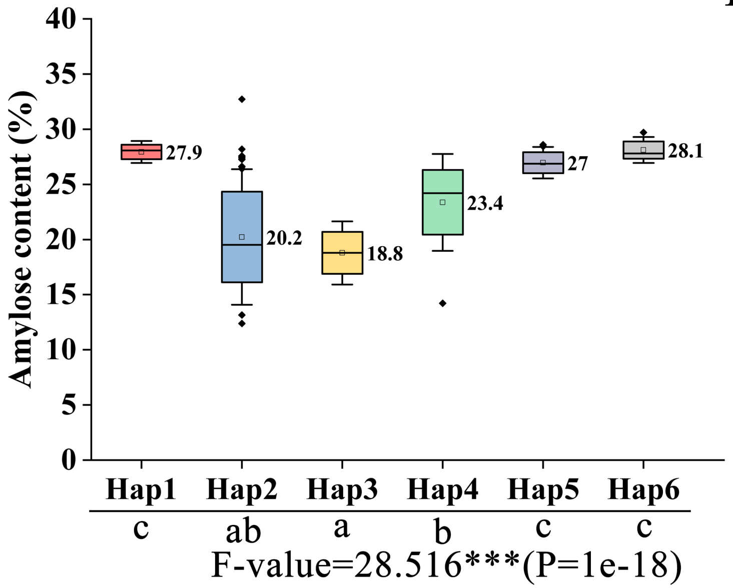

D

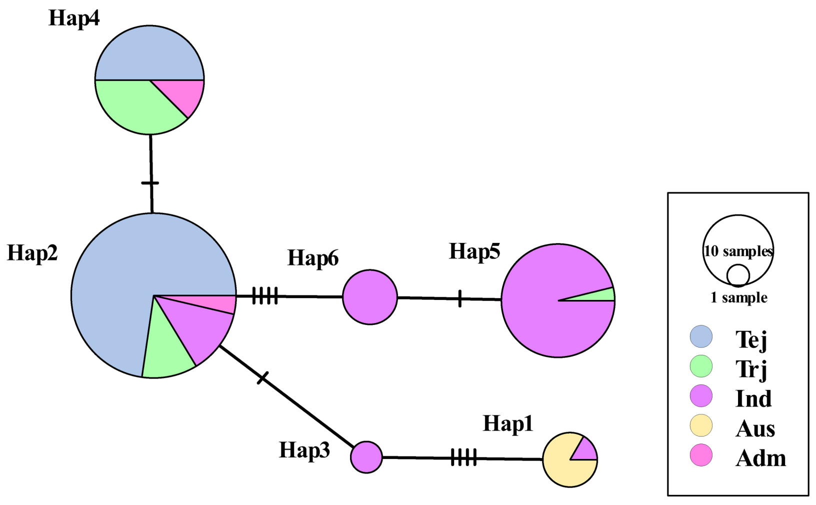

Supplement: Supplementary file 1 [file plants-10-01154-s001.zip › Fig S6.pdf]
