# Supplementary material for: Genome-Wide Identification and Genetic Variations of the Starch Synthase Gene Family in Rice
Source: Plants (Basel). 2021 Jun 6;10(6):1154. doi: 10.3390/plants10061154 (PMC8227427; doi:10.3390/plants10061154)

A

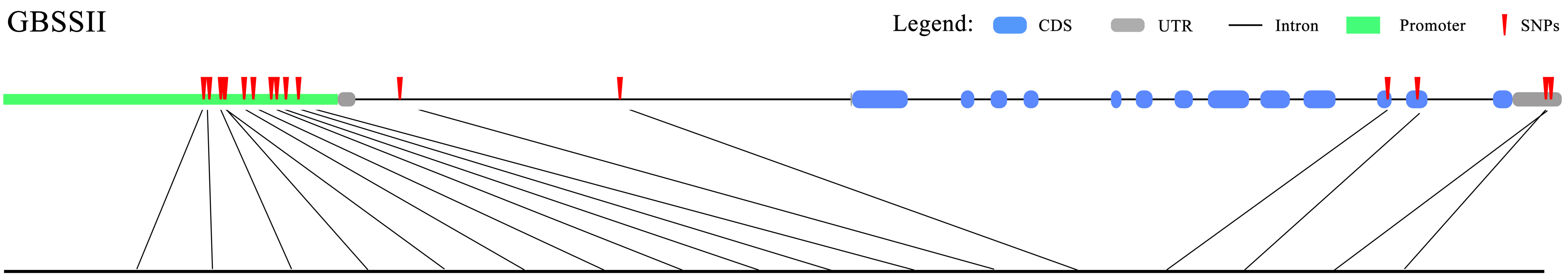

B

ID	SNP 1	SNP 2	SNP 3	SNP 4	SNP 5	SNP 6	SNP 7	SNP 8	SNP 9	SNP 10	SNP 11	SNP 12	SNP 13	SNP 14	SNP 15	SNP 16	SNP 17	Number of accessions
**Region**	Promoter	Promoter	Promoter	Promoter	Promoter	Promoter	Promoter	Promoter	Promoter	Promoter	Promoter	Intron	Intron	Exon	Exon	3'UTR	3'UTR	
**Position**	−800	−766	−698	−677	−671	−558	−503	−396	−363	−308	−233	373	1689	6280	6458	7226	7257	
**Allele**	T/G	C/T	C/G	G/A	T/C	T/C	G/A	G/A	T/A	C/A	T/C	C/T	C/G	G/A	T/C	G/T	A/T	
**Ref.seq**	T	C	C	G	T	T	G	G	T	C	T	C	C	G	T	G	A	
**Hap 1**	G	C	C	A	C	C	A	A	A	A	C	T	C	G	T	G	T	3
**Hap 2**	G	C	C	G	T	C	G	G	T	C	T	T	G	G	T	G	A	2
**Hap 3**	G	C	G	A	C	C	A	A	A	A	C	T	C	A	C	G	T	13
**Hap 4**	T	C	C	G	T	T	G	G	T	C	T	T	G	G	T	T	A	39
**Hap 5**	T	C	C	G	T	T	G	G	T	C	T	T	G	G	T	G	A	43
**Hap 6**	T	T	C	G	T	T	G	G	T	C	T	C	G	G	T	T	A	21

C

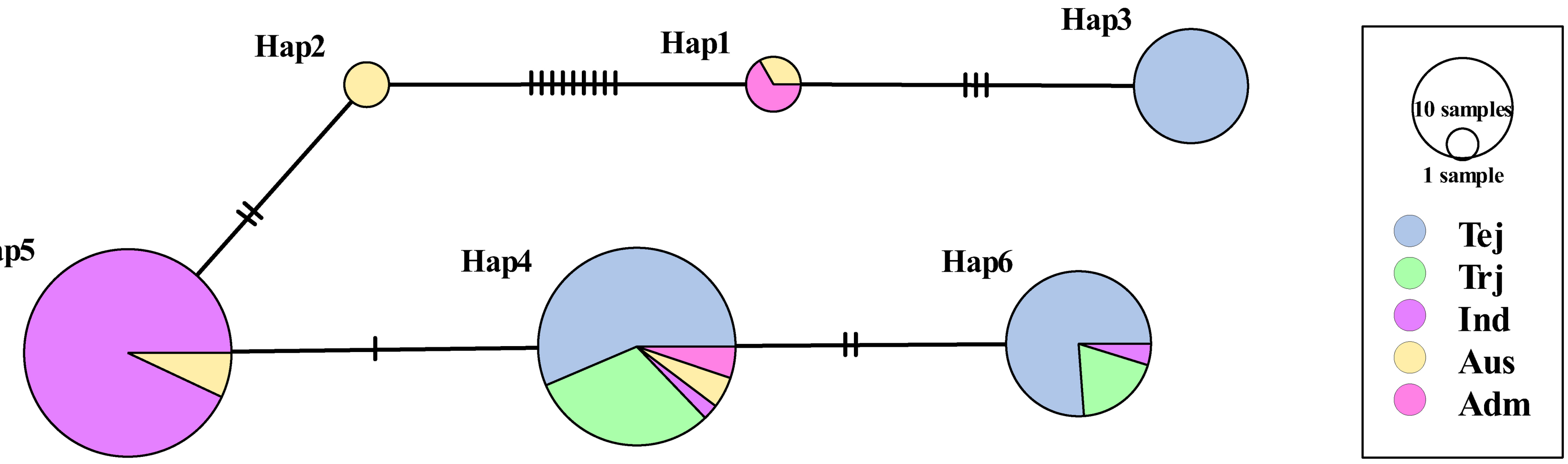

D

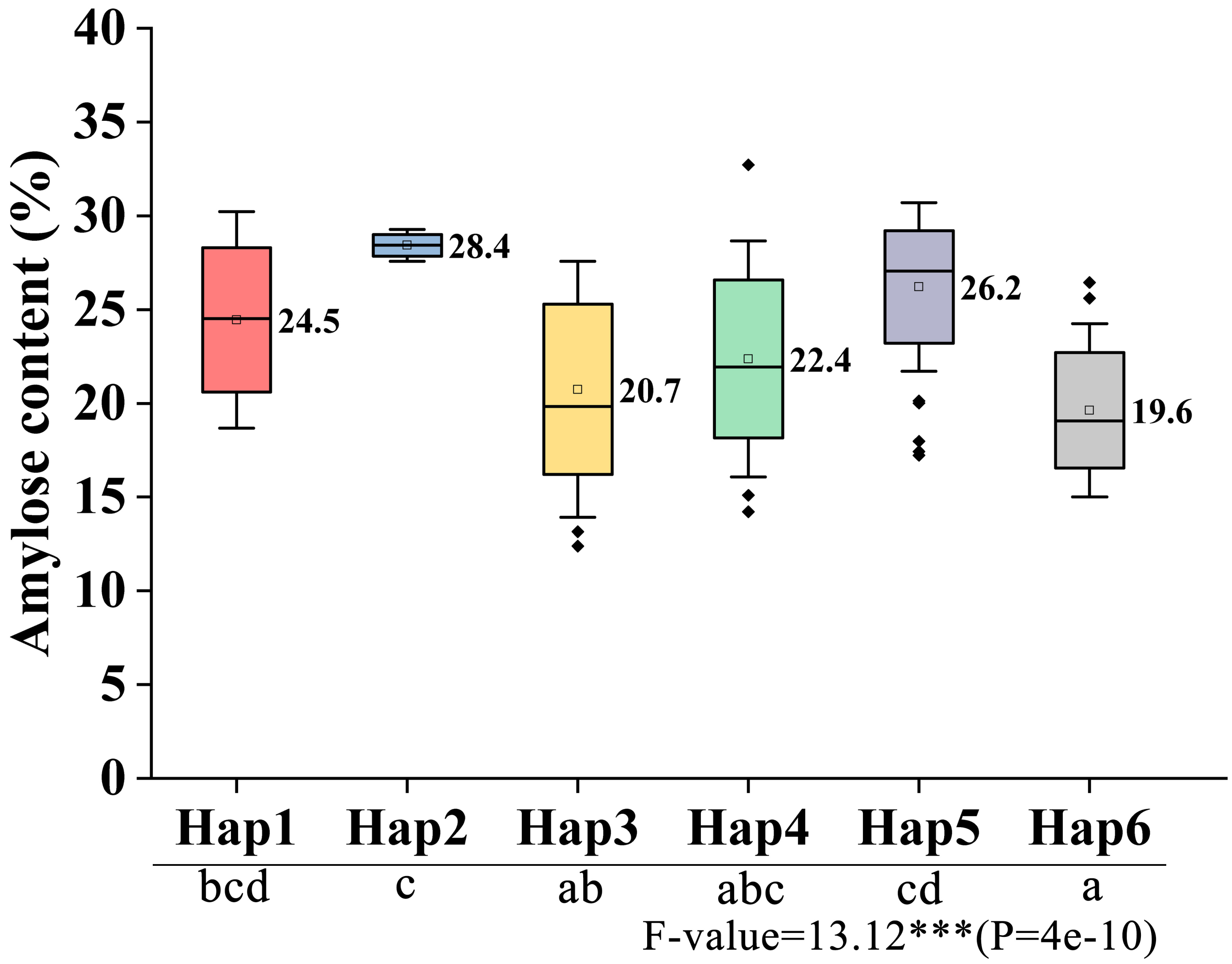

E

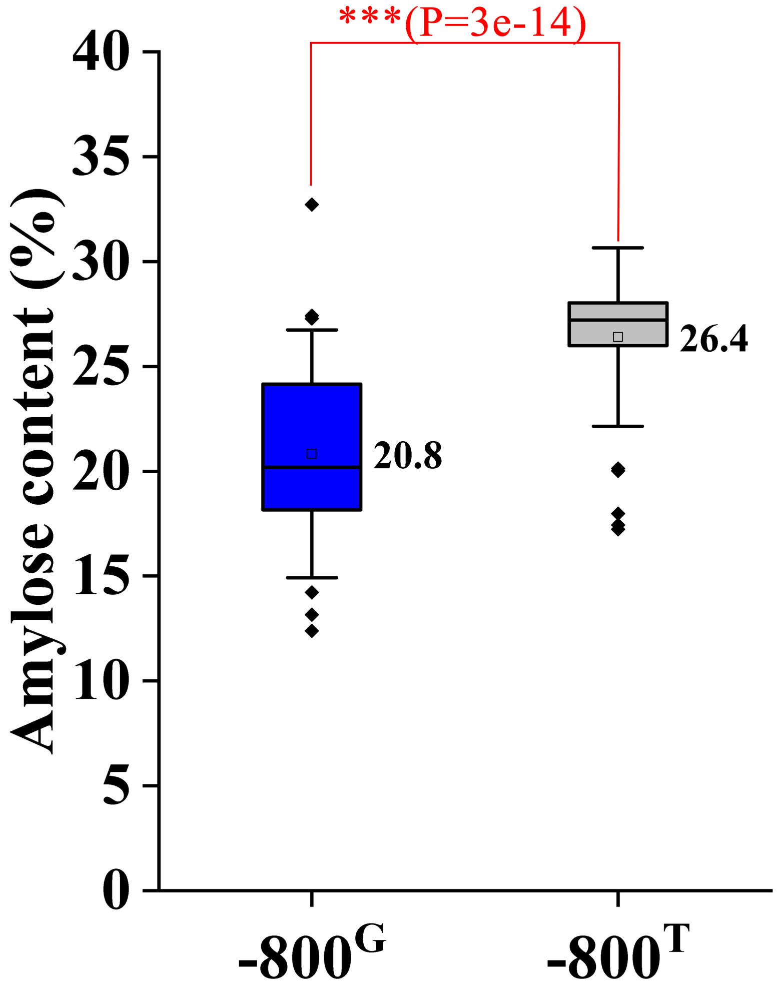

Supplement: Supplementary file 1 [file plants-10-01154-s001.zip › Fig S7.pdf]
