# Supplementary material for: Genome-Wide Identification and Genetic Variations of the Starch Synthase Gene Family in Rice
Source: Plants (Basel). 2021 Jun 6;10(6):1154. doi: 10.3390/plants10061154 (PMC8227427; doi:10.3390/plants10061154)
Supplement: Supplementary file 1 [file plants-10-01154-s001.zip › Starch-supplement Fig.pdf]

Figure S1. Synteny analysis of SSs between rice and other genomes. Each rice chromosome is displayed in a different color. Duplicated gene pairs are displayed and linked using lines with that color. (**A**) *Arabidopsis*, (**B**) barley, (**C**) maize, (**D**) sorghum, (**E**) soybeans, and (**F**) wheat.

Figure S2. Expression profiles of duplications across different growth stages.

Figure S3. Expression profiles of *OsSS* genes. (**A**) Expression related to specific tissues in the flowering stage. (**B**) Expression related to the *OsbZip58-1* mutant line. (**C**) Expression related to the *gif1* mutant.

Figure S4. Putative miRNA targets of *OsSS* genes. The predicted targets are connected by solid lines.

Figure S5. Haplotype analysis of *OsSSIV-2*. (**A**) Structural representation of *OsSSIV-2* and upstream promoter region. (**B**) *OsSSIV-2* SNPs and haplotype groups in 137 rice accessions. SNP positions are given relative to the start of the 5′UTR. Hap: haplotype. (**C**) Association of phenotype with haplotype; different letters indicate significant AC differences between haplotypes (ANOVA, Duncan test). (**D**) Haplotype network variation of *OsSSIV-2*. The circle size represents the number of accessions in each Hap, and the number of transverse lines between each Hap represents the number of nucleotide variations. (**E**). SNP 2 haplotype relationship with AC. Tej: Temperate Japonica; Trj: Tropical Japonica; Ind: Indica; Aus: Aus; and Adm: Admixture rice varieties. (ANOVA and Student's *t*-test, $^{**}p < 0.01$, $^{***}p < 0.001$.)

Figure S6. Haplotype analysis of *OsGBSSI*. (**A**) Structural representation of *OsGBSSI* and upstream promoter region. (**B**) *OsGBSSI* SNPs and haplotype groups in 137 rice accessions. SNP positions are given relative to the start of the 5′UTR. Hap: haplotype. (**C**) Association of phenotype with haplotype; different letters indicate significant AC differences between haplotypes (ANOVA, Duncan test). (**D**) Haplotype network variation of *OsGBSSI*. The circle size represents the number of accessions in each Hap, and the number of transverse lines between each Hap represents the number of nucleotide variations. (ANOVA, $^{***}p < 0.001$).

Figure S7. Haplotype analysis of *OsGBSSII*. (**A**) Structural representation of *OsGBSSII* and upstream

promoter region. (**B**) *OsGBSSII* SNPs and haplotype groups in 137 rice accessions. SNP positions are given relative to the start of the 5′UTR. Hap: haplotype. (**C**) Association of phenotype with haplotype; different letters indicate significant AC differences between haplotypes (ANOVA, Duncan test). (**D**) Haplotype network variation of *OsGBSSII*. The circle size represents the number of accessions in each Hap, and the number of transverse lines between each Hap represents the number of nucleotide variations. (**E**) SNP 1 haplotype relationship with AC. Tej: Temperate *Japonica*; Trj: Tropical *Japonica*; Ind: *Indica*; Aus: Aus; and Adm: Admixture rice varieties. (ANOVA and Student's *t*-test, ***$p < 0.001$).
