# Supplementary figures and images for: Genome-Wide Identification and Genetic Variations of the Starch Synthase Gene Family in Rice
Source: Plants (Basel). 2021 Jun 6;10(6):1154. doi: 10.3390/plants10061154 (PMC8227427; doi:10.3390/plants10061154)

A

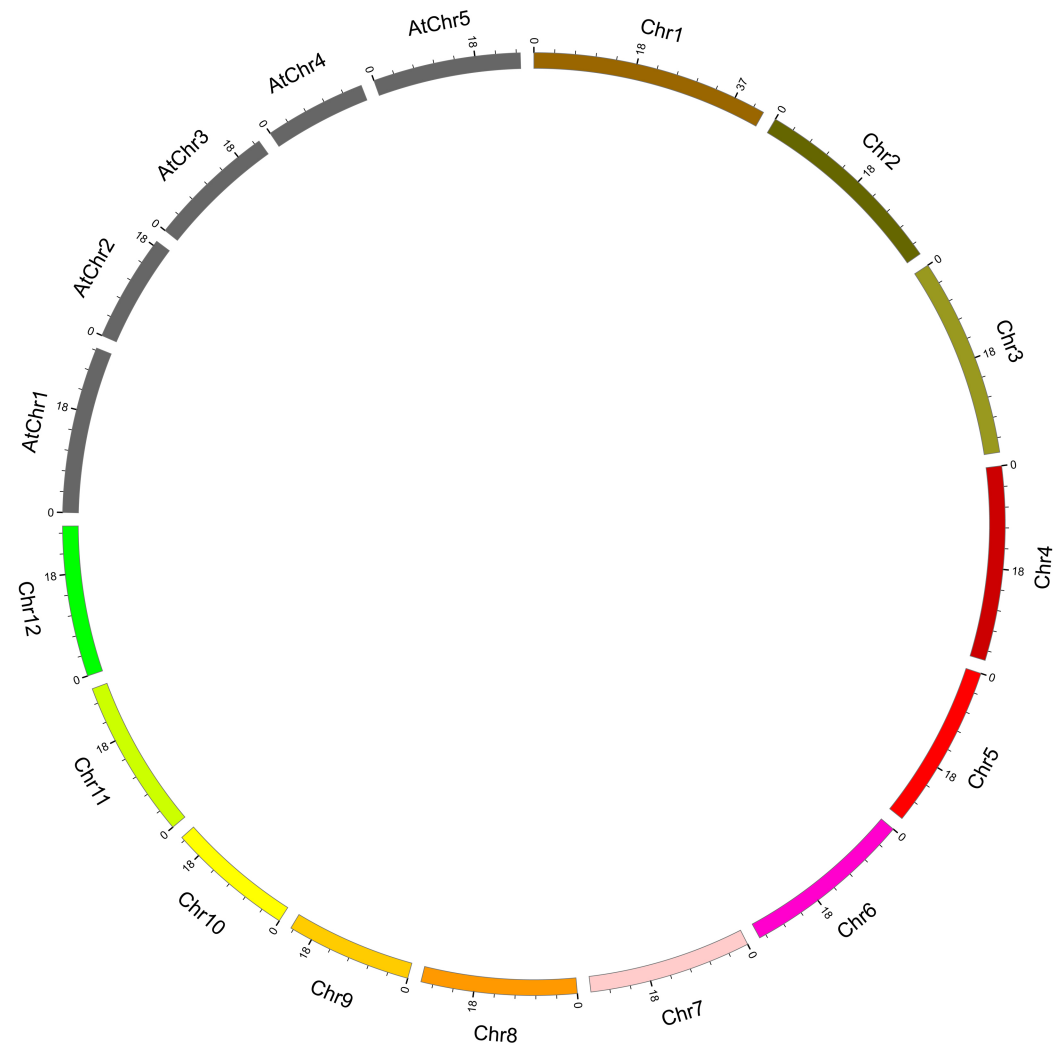

B

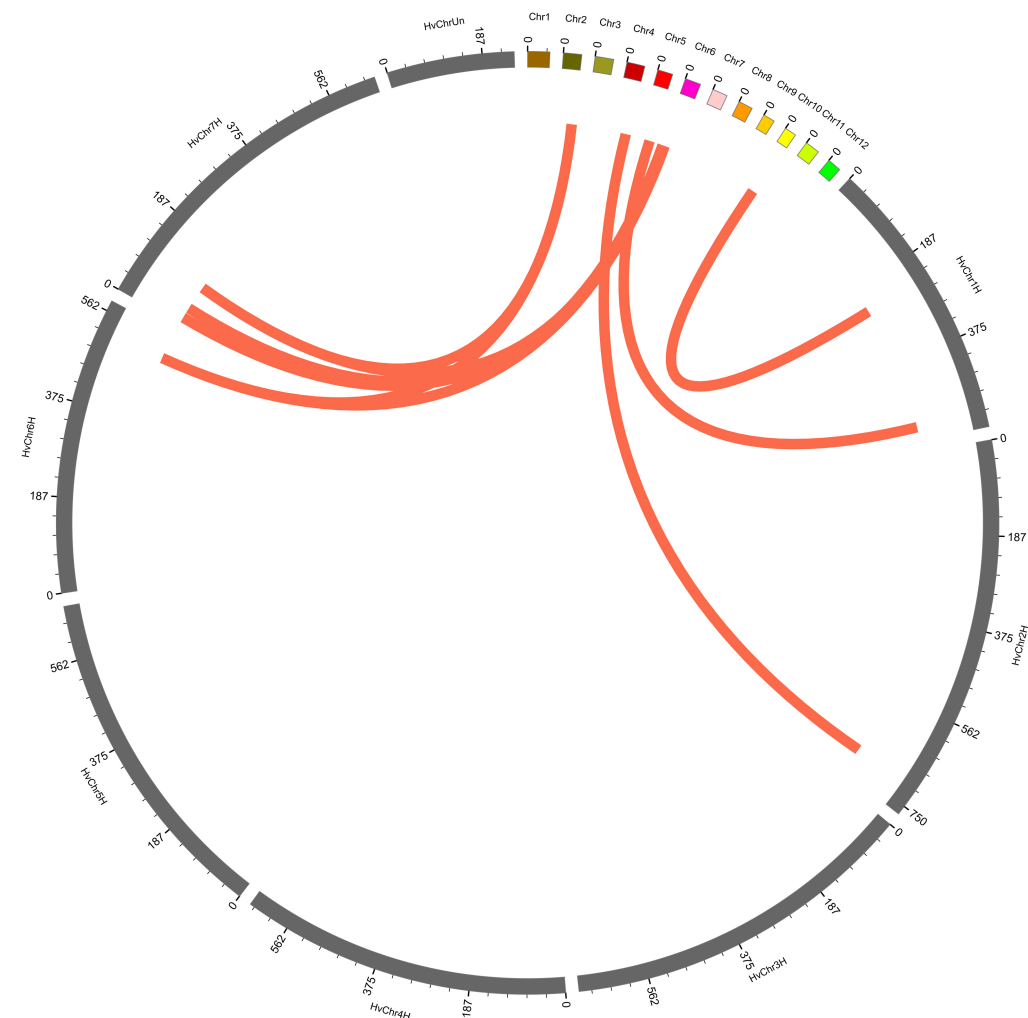

C

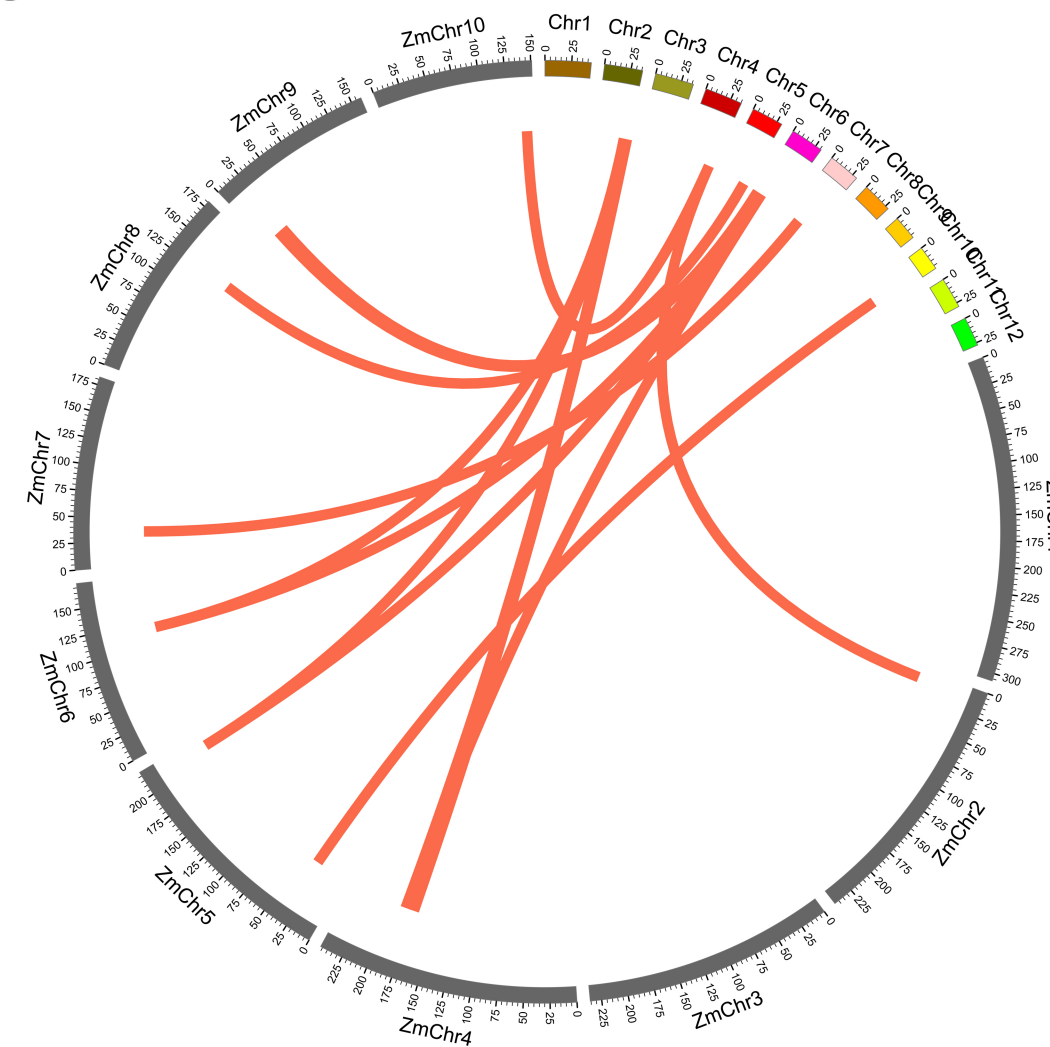

D

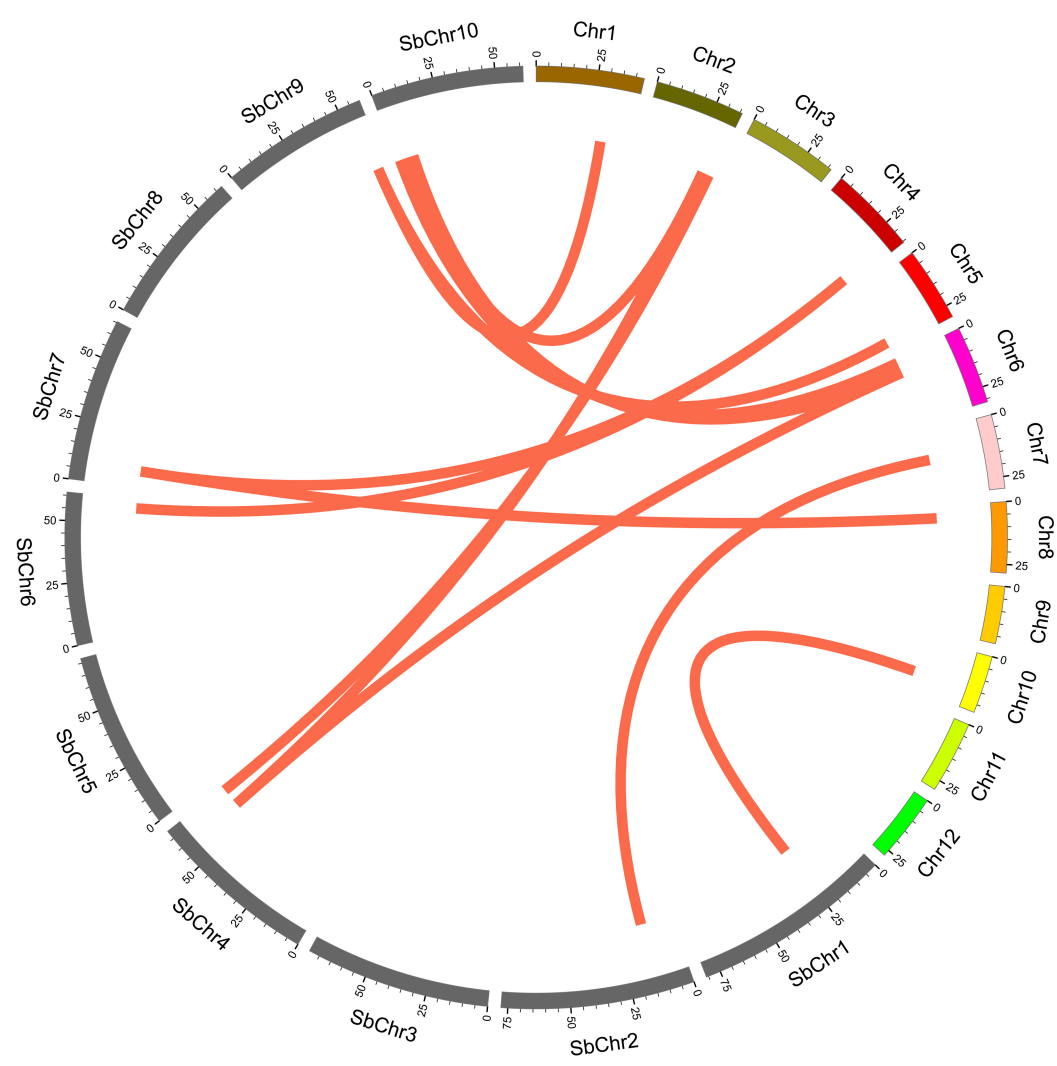

E

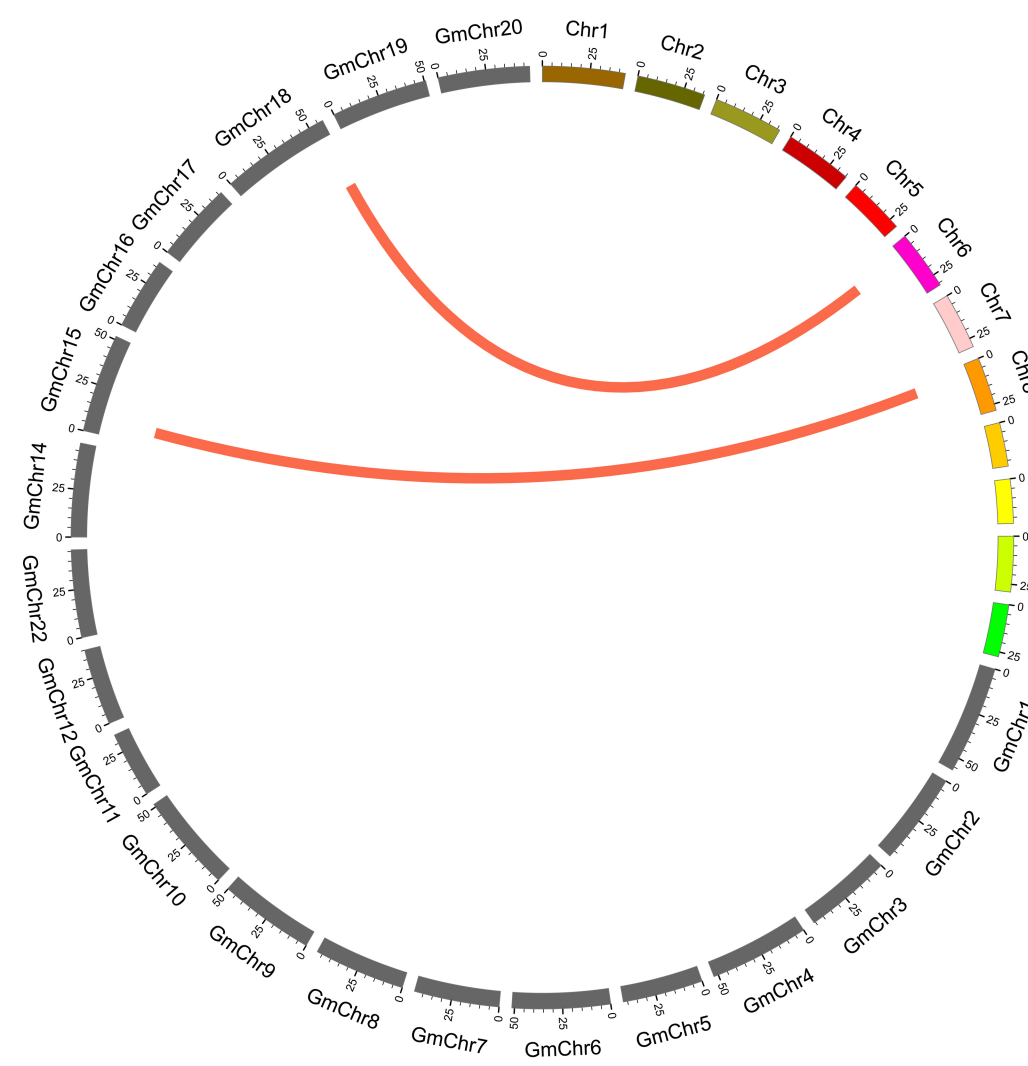

F

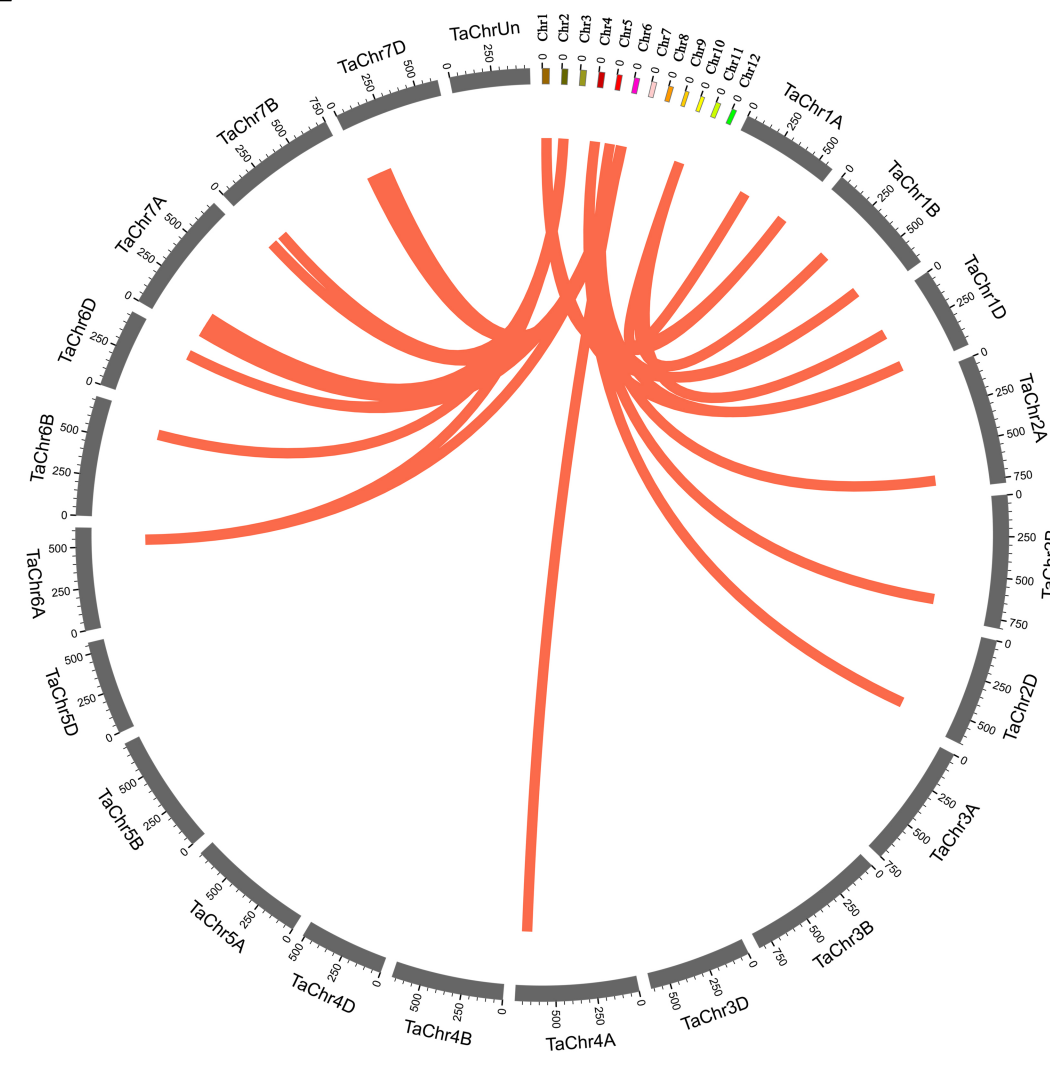

Supplement: Supplementary file 1 [file plants-10-01154-s001.zip › Fig S1.pdf]

A

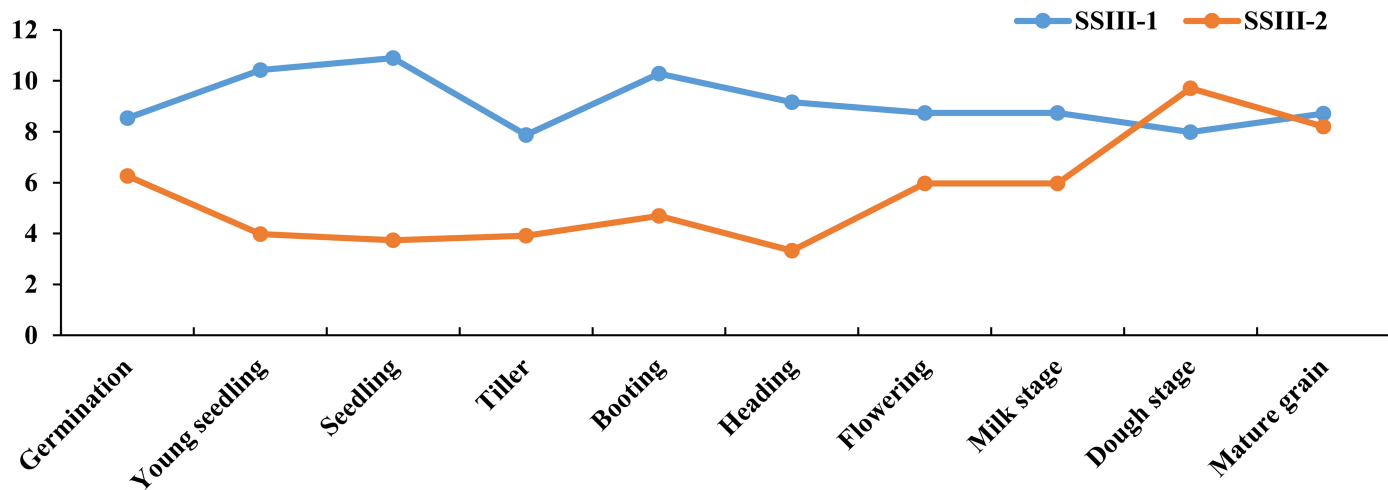

B

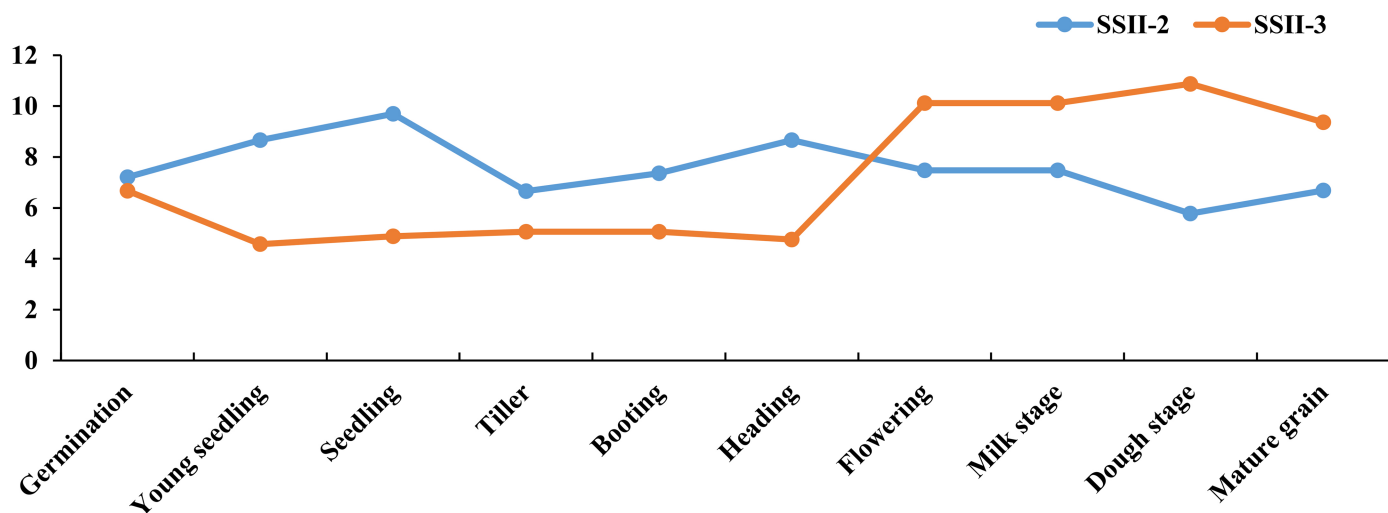

C

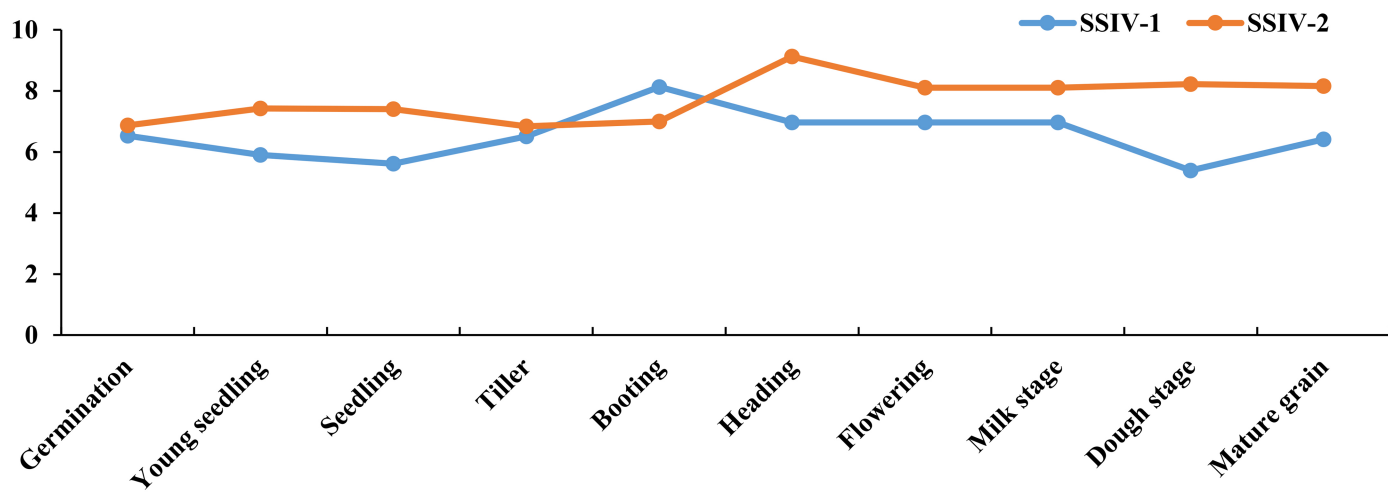

Supplement: Supplementary file 1 [file plants-10-01154-s001.zip › Fig S2.pdf]

A

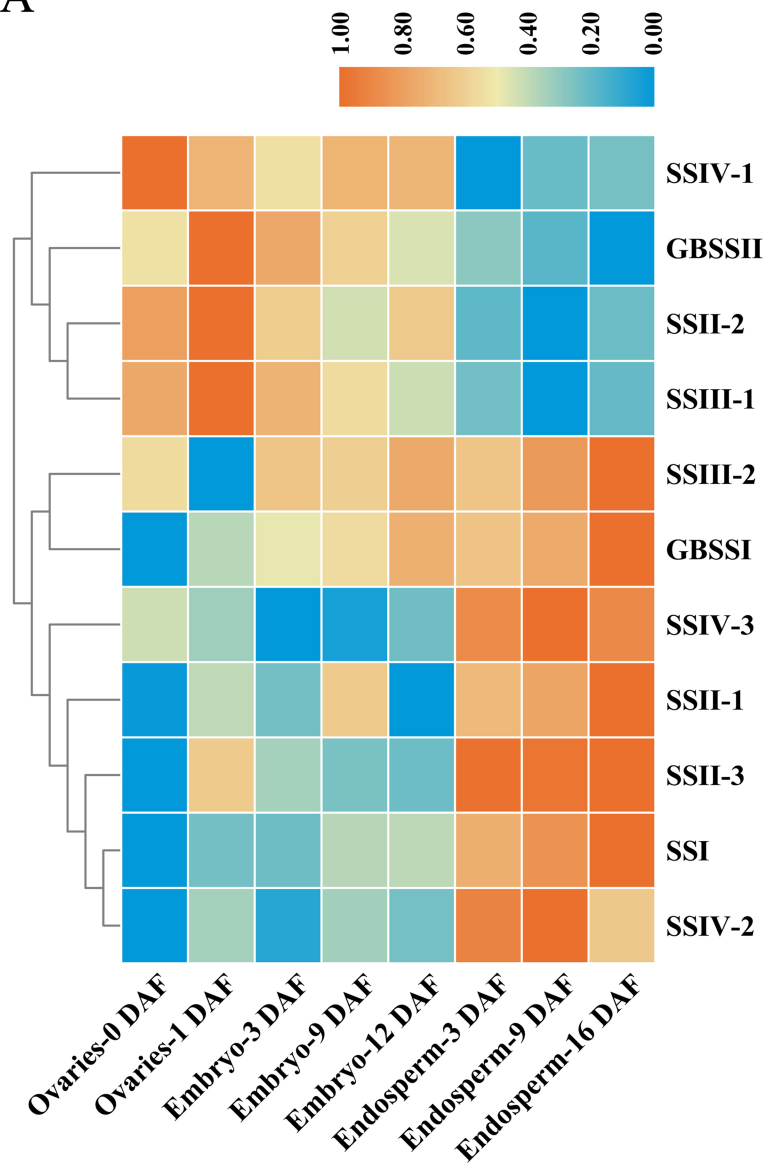

B

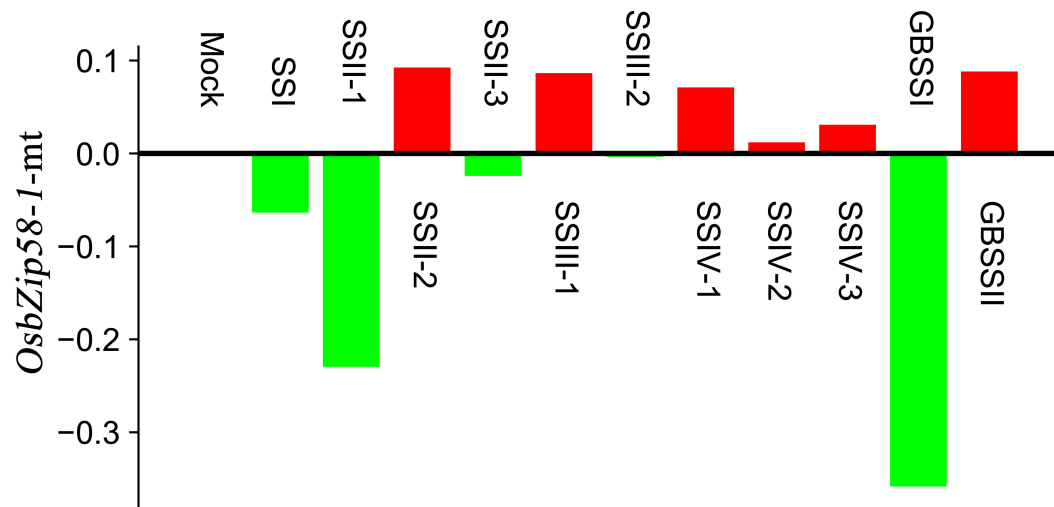

C

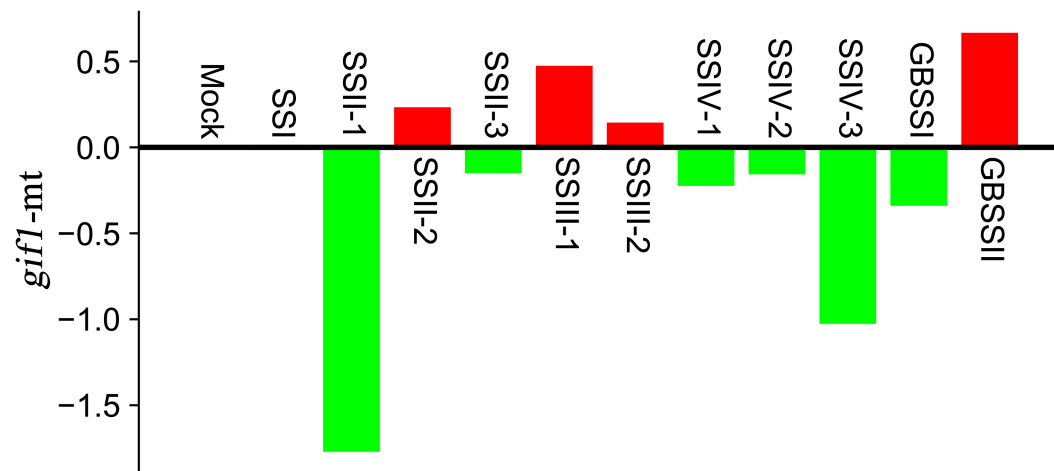

Supplement: Supplementary file 1 [file plants-10-01154-s001.zip › Fig S3.pdf]

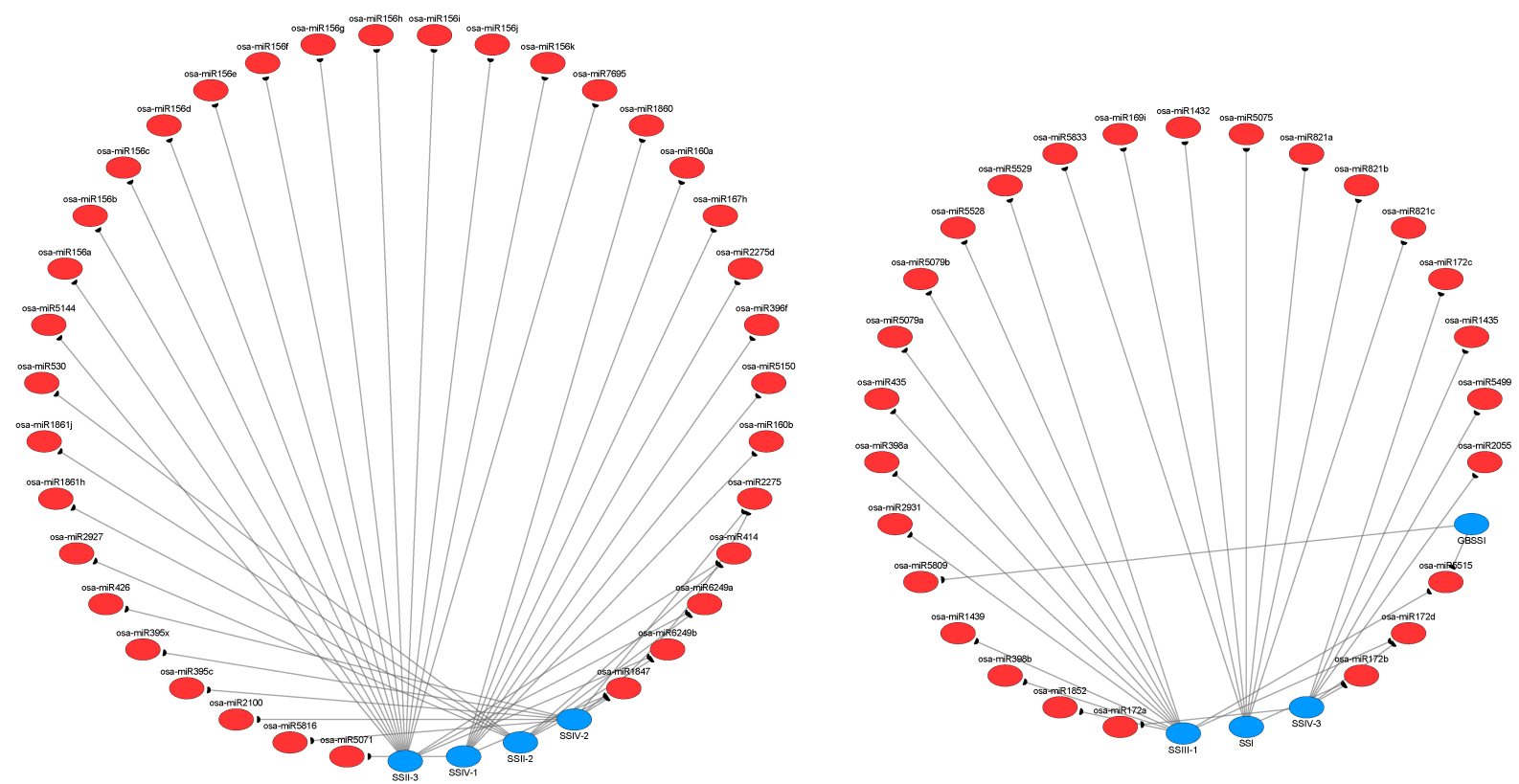

Supplement: Supplementary file 1 [file plants-10-01154-s001.zip › Fig S4.pdf]
